# Biochemical and Structural Characterization of Thermostable GH159 Glycoside Hydrolases Exhibiting α-L-Arabinofuranosidase Activity

**DOI:** 10.3389/fmolb.2022.907439

**Published:** 2022-06-29

**Authors:** Melanie Baudrexl, Tarik Fida, Berkay Berk, Wolfgang H. Schwarz, Vladimir V. Zverlov, Michael Groll, Wolfgang Liebl

**Affiliations:** ^1^ Chair of Microbiology, Technical University of Munich, Freising, Germany; ^2^ Institute of Molecular Genetics, Russian Academy of Science, Moscow, Russia; ^3^ Chair of Biochemistry, Center for Protein Assemblies, Technical University of Munich, Garching, Germany

**Keywords:** β-D-galactofuranosidase, α-L-arabinofuranosidase, glycoside hydrolase family GH159, thermophilic, *Caldicellulosiruptor*, crystallographic structure analysis, five-bladed beta propeller, active site

## Abstract

Functional, biochemical, and preliminary structural properties are reported for three glycoside hydrolases of the recently described glycoside hydrolase (GH) family 159. The genes were cloned from the genomic sequences of different *Caldicellulosiruptor* strains. This study extends the spectrum of functions of GH159 enzymes. The only activity previously reported for GH159 was hydrolytic activity on β-galactofuranosides. Activity screening using a set of *para*-nitrophenyl (*p*NP) glycosides suggested additional arabinosidase activity on substrates with arabinosyl residues, which has not been previously reported for members of GH159. Even though the thermophilic enzymes investigated—*Cs_*Gaf159A, *Ch_*Gaf159A, and *Ck*_Gaf159A—cleaved *p*NP-α-l-arabinofuranoside, they were only weakly active on arabinogalactan, and they did not cleave arabinose from arabinan, arabinoxylan, or gum arabic. However, the enzymes were able to hydrolyze the α-1,3-linkage in different arabinoxylan-derived oligosaccharides (AXOS) with arabinosylated xylose at the non-reducing end (A^3^X, A^2,3^XX), suggesting their role in the intracellular hydrolysis of oligosaccharides. Crystallization and structural analysis of the apo form of one of the *Caldicellulosiruptor* enzymes, *Ch*_Gaf159A, enabled the elucidation of the first 3D structure of a GH159 member. This work revealed a five-bladed β-propeller structure for GH159 enzymes. The 3D structure and its substrate-binding pocket also provides an explanation at the molecular level for the observed exo-activity of the enzyme. Furthermore, the structural data enabled the prediction of the catalytic amino acids. This was supported by the complete inactivation by mutation of residues D19, D142, and E190 of *Ch*_Gaf159A.

## Introduction

Carbohydrates are the most common and, due to their structural heterogeneity, one of the most diverse classes of biopolymers (Pauly and Keegstra 2008). Their hydrolysis depends on the simultaneous presence of a wide range of different enzymatic activities. These Carbohydrate Active Enzymes (CAZymes) are, like their substrates, incredibly diverse. At the same time, each enzyme is highly specific for both the type of sugar bound and its glycosidic bond. This is also reflected in the large number of sequences in the CAZy database (Lombard et al., 2014), which classifies these enzymes, according to sequence similarities and conformity of 3D structure, into clans and families. Currently 18 GH clans of related GH families and seven different folding types can be distinguished, among which are mainly barrel-formed 3D structures (http://www.cazy.org/Glycoside-Hydrolases.html). Small variations in the catalytic center of an enzyme can cause a different substrate specificity, which is why most glycoside hydrolase (GH) families contain enzymes with different catalytic functions. In contrast, the Enzyme Commission (E.C.) number indicates which chemical reaction is catalyzed by an enzyme, irrespective of the enzyme’s structure. Accordingly, enzymes with two different activities are assigned two E.C. numbers.

Helbert et al. (2019) experimentally confirmed β-d-galactofuranosidase (EC 3.2.1.146; Gaf) activity for BcellWH2_02356 (ALJ59596.1) from *Bacteroides cellulosilyticus* WH2 using heterologously produced enzyme and the artificial substrate *para*-nitrophenyl-β-d-galactofuranoside (*p*NPG), whereas oligo- or polysaccharides have not been tested as substrates. Due to the lack of similarity to previously described GH families, this enzyme became the founding member of the new GH family GH159. Since 2019, some formerly unclassified glycoside hydrolase (GHnc) sequences have been assigned to GH159 on the basis of amino acid sequence similarities, including putative enzymes encoded on the genomes of different *Caldicellulosiruptor* strains. The members of the genus *Caldicellulosiruptor* are extremely thermophilic, cellulolytic, and non-spore-forming flagellated strict anaerobes with Gram-positive type cell walls, and are capable of fermenting a wide spectrum of carbohydrates. *Caldicellulosiruptor saccharolyticus* was isolated from wood in the flow from a geothermal spring in New Zealand and has a growth range of between 45°C and 80°C at pH 5.5 to 8.0 (optimum at 70°C and pH 7.0) (Rainey et al., 1994). *Caldicellulosiruptor hydrothermalis* grows between 50 and 80°C at pH 6.0 to 8.0 with optimum growth at 65°C and pH 7.0. The same growth properties were found for *Caldicellulosiruptor kronotskyensis*, but the temperature range (45–82°C) is broader and the optimum higher (70°C) (Miroshnichenko et al., 2008).

In this study, the hitherto uncharacterized ORFs encoding three GH159 enzymes, *Cs*_Gaf159A, *Ch*_Gaf159A, and *Ck*_Gaf159A with similar sequences were cloned from the three aforementioned *Caldicellulosiruptor* species *C. saccharolyticus*, *C. hydrothermalis* and *C. kronotskyensis* with the aim of biochemically characterizing the heterologously expressed enzymes with regard to substrate specificity, temperature and pH dependence of activity, their stability at elevated temperatures, the effects of metal ions, and inhibition by products. In order to characterize the substrate binding and catalytic mechanism of enzymes in more detail at the molecular level from the previously (poorly) studied GH159 family, for which no three-dimensional structures were available, we also sought to elucidate the 3D structure of one of the enzymes by means of crystallography.

## Results

### Cloning, Expression, and Purification

*Caldicellulosiruptor* strains *C. saccharolyticus* DSM 8903, *C. hydrothermalis* 108 (DSMZ 18901), and *C. kronotskyensis* 2002 (DSMZ 18902) were grown anaerobically, and genomic DNA was isolated in order to amplify the sequences of the genes with locus tags Csac_0437 (ABP6075.1), Calhy_0274 (ADQ06027.1) and Calkro_0290 (ADQ45201), respectively.

**FIGURE 1:**
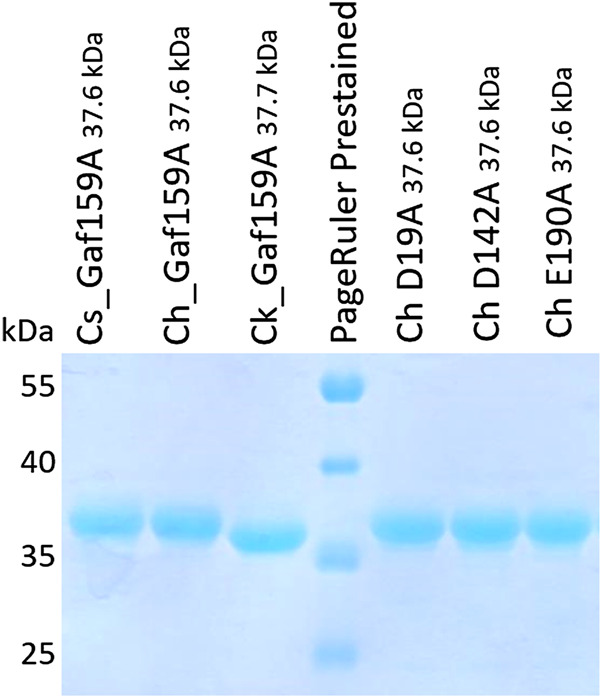
SDS-PAGE analysis of recombinantly produced wildtype and mutant enzymes. Protein samples (0.5 µg μl^−1^) were mixed with 4 x SDS-loading buffer (0.25 M Tris, 40% w/v glycerol, 0.29 M SDS, 0,57 β-mercaptoethanol, 0.02% bromophenol blue, HCl to pH 6.8), 10 µl were loaded into each lane. Size markers were applied as PageRuler™ Prestained Protein Ladder, 10–180 kDa.

After cloning in expression vector pET24c(+), the expected constructs were verified by sequencing. Expression in *E. coli* BL21 Star and the subsequent purification resulted in sufficient amounts of the proteins for further study. Separation by SDS-PAGE showed bands corresponding to the expected molecular weights including the His_6_-tags (*Cs_*Gaf159A: 37.6 kDa; *Ch_*Gaf159A: 37.6 kDa; *Ck_*Gaf159A: 37.7 kDa) for all enzyme preparations used in this study (Figure 1). A high degree of purity as judged from SDS-PAGE was achieved by means of immobilized metal affinity chromatography. Heat treatment of crude extracts from the recombinant *E. coli* strains at 55°C for depletion of host proteins did not result in a much higher purity, although only small amounts of the respective enzymes precipitated (Supplementary Figure S1).

### Characterization Using *para*-Nitrophenyl Glycoside Substrates

**FIGURE 2:**
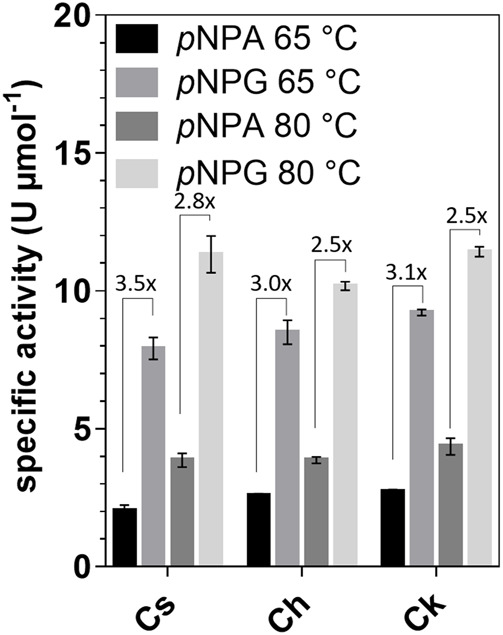
Specific activities of *Cs*_Gaf195A, *Ch*_Gaf159A and *Ck*_Gaf159A with *p*NPA and *p*NPG at 65 and 80°C. The *p*NPG-cleaving and *p*NPA-cleaving activities are indicated as fold changes. Standard reactions contained 0.1 µg μl^−1^ enzyme and were performed in triplicates and incubated for 20 min.

Activity assays with *para*-nitrophenyl-β-d-galactofuranoside (*p*NPG), *p*NP-α-l-arabinofuranoside (*p*NPA), *p*NP-α-d-galactopyranoside, *p*NP-α-l-fucopyranoside, *p*NP-β-l-fucopyranoside, *p*NP-β-d-glucopyranoside, *p*NP-β-d-xylopyranoside, *p*NP-β-d-mannopyranoside, *p*NP-α-l-rhamnopyranoside, and *p*NP-α-d-glucopyranosiduronic acid as substrates showed that the enzymes were active only on *p*NPG and *p*NPA. The activity with *p*NPG was on average 3.6-fold higher at 65°C and 2.6-fold higher at 80°C than the activity with *p*NPA (Figure 2). K_m_ values with *p*NPG ranged from 4.2 to 8.5 mM, while the maximum velocity (V_max_) was determined to be 2.7–2.9 U mg^−1^ (Supplementary Figure S2). Therefore, the enzymes can be classified as galactofuranosidases (Gafs).

**FIGURE 3:**
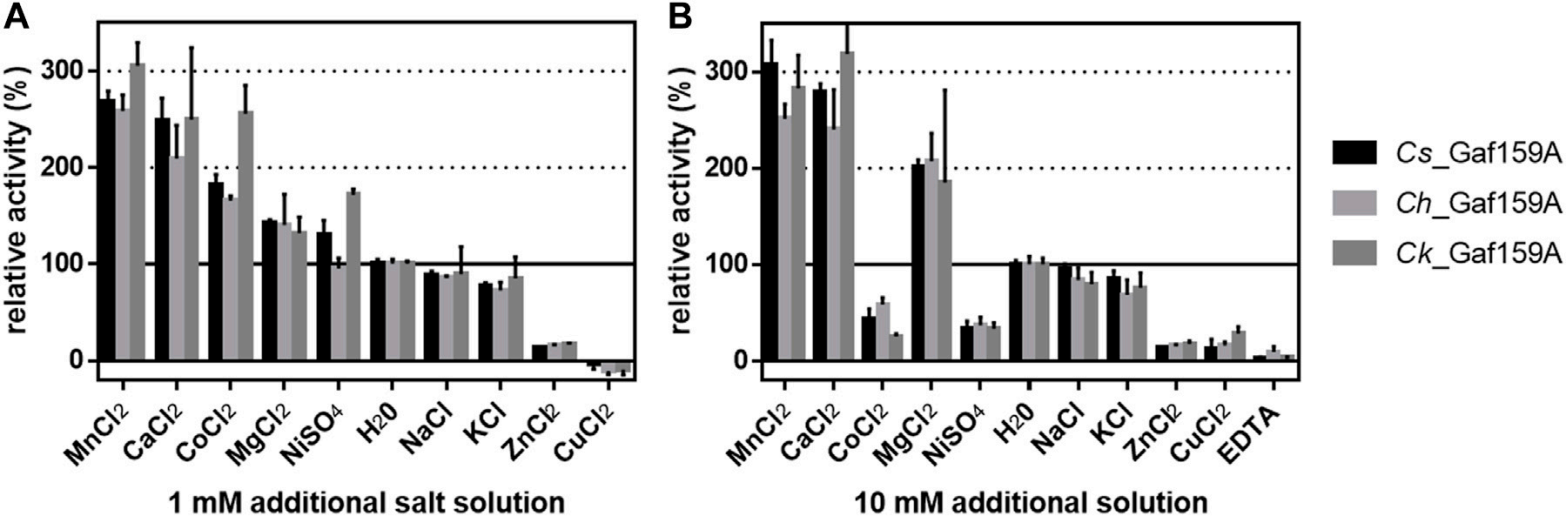
Influence of additional salt solutions on the activity of *Cs*_Gaf159A, *Ch*_Gaf159A and *Ck*_Gaf159 on *p*NPA. Reactions contained 1 mM pNPA, 5 µg enzyme (0.2 µg μl^−1^ stocks stored in 0.1 M MOPS pH 6.5), 0.1 M MOPS pH 6.5, and either 1 mM **(A)**, or 10 mM **(B)** additional salt solutions, and were incubated for 20 min at the respective temperature optima (90, 82 and 93°C for *Cs*, *Ch* and *Ck*). Bars represent standard deviation from triplicates.

Since arabinofuranosidase activity was not previously reported for GH159 enzymes, we mainly focused on this newly detected activity in the present work. The mean specific activities on *p*NPA for *Cs*_Gaf159A, *Ch*_Gaf159A, and *Ck*_Gaf159A were 0.04, 0.04, and 0.03 U mg^−1^, which corresponds to 1.5, 1.5 and 1.3 U µmol^−1^, all respectively. To uncover the influence of different metal ions on the activity, standard assay reactions were supplemented with various salt solutions. The addition of 1 mM of MnCl_2,_ CaCl_2_, MgCl_2_, and CoCl_2_ increased the activity (see Supplementary Table S1). NaCl and KCl had nearly no or a slightly negative effect, whereas addition of 1 mM ZnCl_2_ or CuCl_2_ reduced the activity by 80% (Figure 3A). At 10 mM, the effects of the salts on enzymes activity were similar, the difference being that the higher concentrations of NiSO_4_ and CoCl_2_ had an inhibitory effect (Figure 3B). The data for MnCl_2_, CoCl_2_, NiCl_2_, and CuCl_2_ that is shown in Figure 3 should not be overinterpreted due to color and precipitate formation under the assay conditions and hence interference with the assay used. Based on its activity-enhancing effect, 10 mM CaCl_2_ was included in the standard assay buffer. This led to at least a two-fold increase in specific activities, i.e., about 0.1 U mg^−1^ (3.76 U µmol^−1^), 0.09 U mg^−1^ (3.39 U µmol^−1^), and 0.08 U mg^−1^ (3.02 U µmol^−1^) with *p*NPA as substrate, of *Cs*-, *Ch*- and *Ck*_Gaf159A, respectively. Addition of 10 mM of the chelating agent EDTA resulted in an almost complete inactivation of all three enzymes, thus confirming their dependency on divalent metal ions (Figure 3B).

**FIGURE 4:**
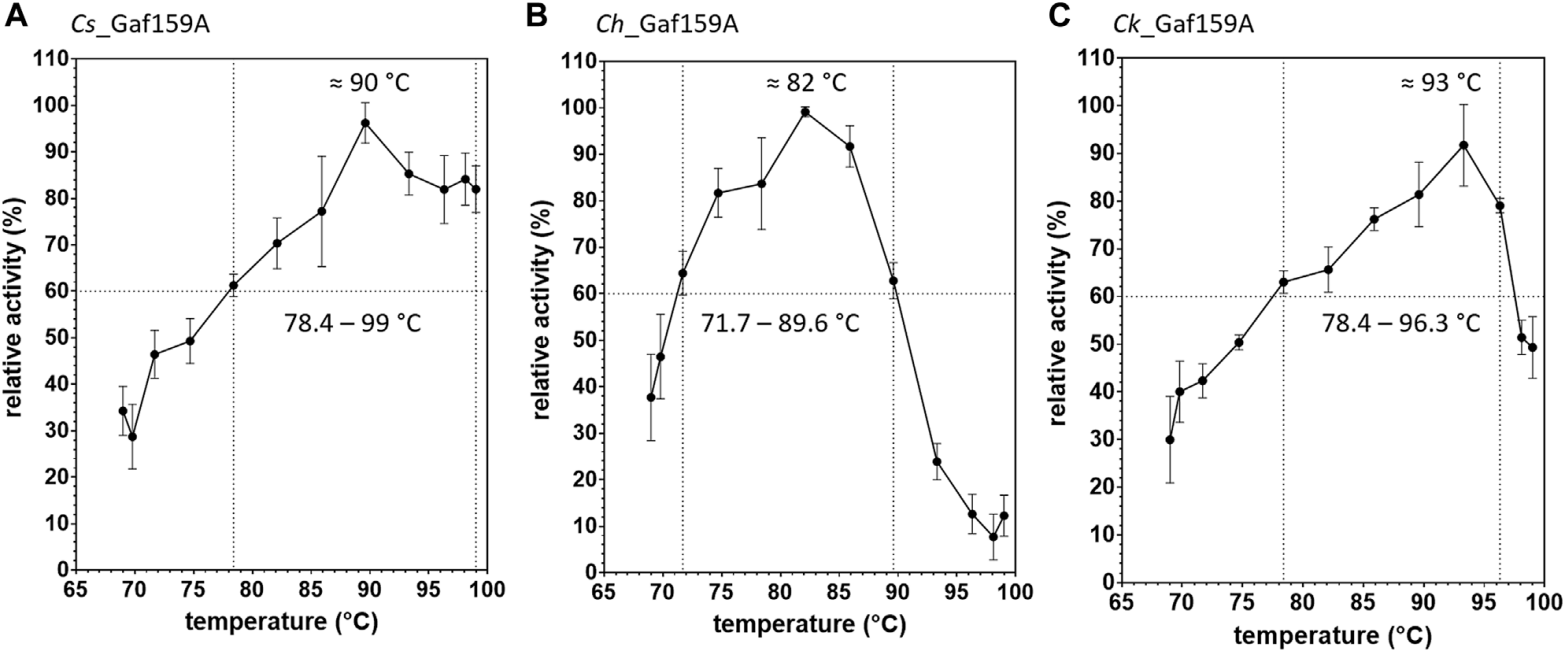
Effect of temperature on pNPAase activity of *Cs*_Gaf159A **(A)**, *Ch*_Gaf159A **(B)** and *Ck*_Gaf159A **(C)**. Relative activities to the maximal activity measured after 20 min were calculated from *p*NP standard reactions (2.65 µM enzyme). Bars represent standard deviation from triplicates.

The temperature ranges in which the enzymes displayed at least 60% activity (pH 6.5) were 78.4–99.0°C for *Cs_*Gaf159A, 71.7–89.6°C for *Ch_*Gaf159A and 78.4–96.3°C for *Ck_*Gaf159A, whereas the temperatures for maximal activity (20 min assay with *p*NPA as substrate) were 90°C, 82°C, and 93°C, respectively (see Figure 4).

**FIGURE 5:**
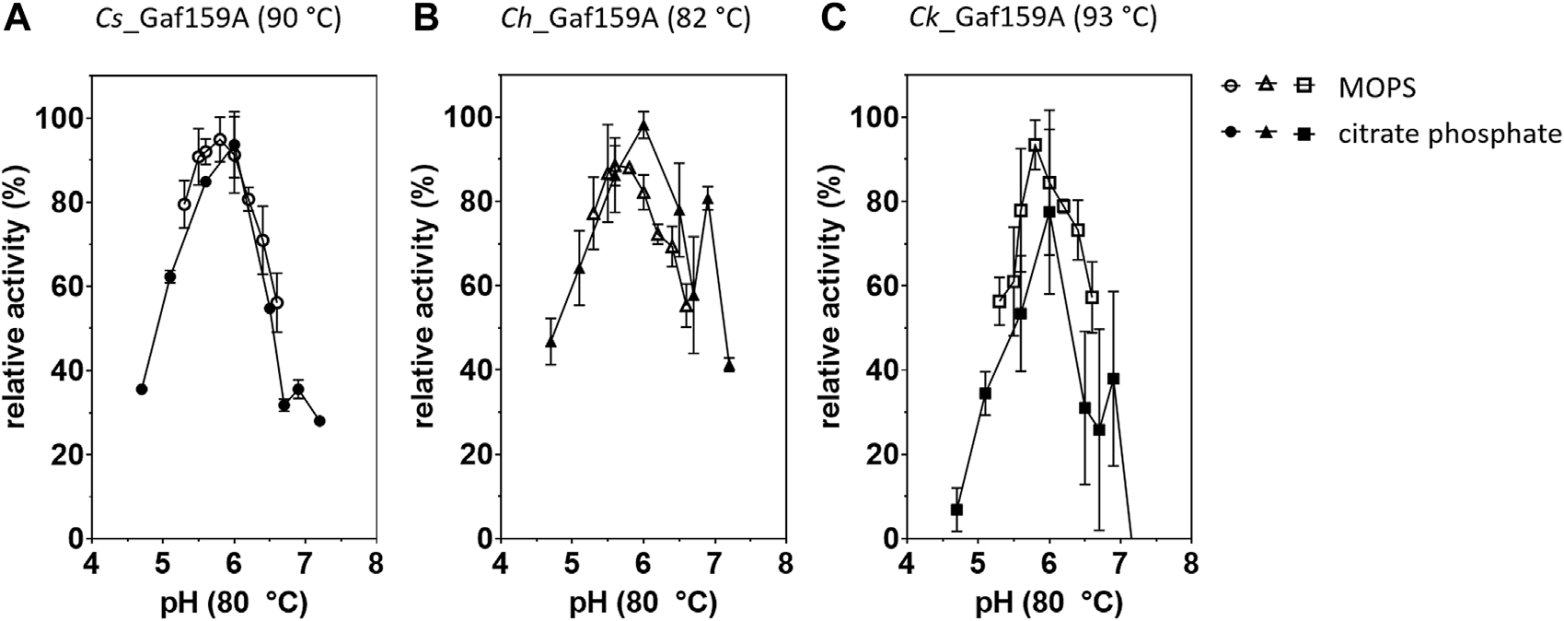
Influence of different pH values on the activity of *Cs*-, *Ch*- and *Ck*_Gaf159A. Reactions contained either McIlvaine (filled symbols), or MOPS buffer (empty symbols), 2.65 µM enzyme (0.1 µg μl^−1^), 1 mM pNPA, 1 x RP and 1 mM CaCl_2_ and were incubated for 20 min at the respective temperature optimum. **(A)** *Cs*_Gaf159A, 90°C; **(B)** *Ch*_Gaf159A, 82°C; **(C)** *Ck*_Gaf159A, 93°C. Activities are related to highest activity with the respective buffer. Bars represent standard deviation from triplicates.

The pH optimum, as determined at the respective apparent 20 min temperature optimum of each enzyme, differed less between the enzymes than the temperature optima. Using assays carried out at 80°C all three Gafs displayed the highest activity at around pH 5.6–6 (Figure 5). At room temperature, the optimal pH levels were found to be at pH 5.5–6 in McIlvaine+ buffer and pH 6.4–6.8 in MOPS buffer (Supplementary Table S2). The specific activities in McIlvaine+ buffer were from two to more than four times lower than in MOPS, with 0.02 U mg^−1^ (0.8 U µmol^−1^) for *Cs_*Gaf159A and 0.03 U mg^−1^ (1.0 U µmol^−1^) for *Ch_*Gaf159A and *Ck_*Gaf159A, possibly due to complex formation between the divalent cations and the phosphate present in the McIlvaine+ buffer system.

**FIGURE 6:**
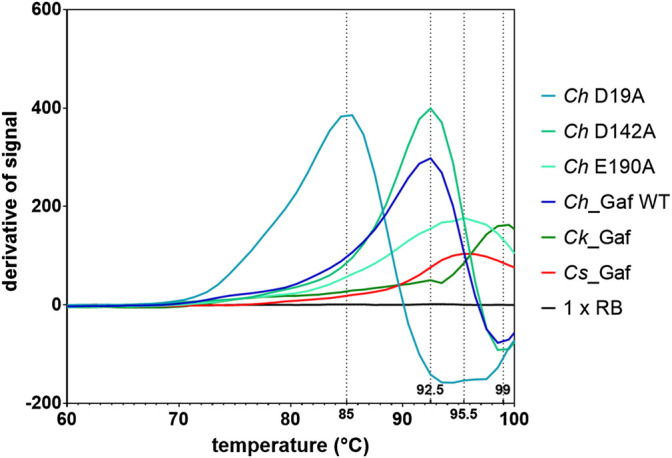
Melting temperatures of *Cs*_Gaf159A, *Ck*_Gaf159A, *Ch*_Gaf159A and the mutants *Ch*_D19A, *Ch*_D142A and *Ch*_E190A according to differential scanning fluorimetry analysis with SYPRO Orange. Smoothed differentiated curves from triplicates.

Regarding resistance against thermo-inactivation, incubation of the enzymes at their apparent “optimal” temperatures (temperature of maximum activity in a 20 min assay with substrate *p*NPA) indicated a high level of thermostability, especially for *Cs_*Gaf159A. Incubation of up to 24 h at 90°C did not seem to affect the activity of the latter. *Ch_*Gaf159A lost 50% of its activity after 5 h at 82°C, and *Ck*_Gaf159A lost nearly 50% of its activity after 2.5 h at 93 °C (Supplementary Figure S3). Although *Cs_*Gaf159A revealed an apparently extraordinary heat stability at 90 °C, variation of the assay time between 10 and 30 min indicated that heat inactivation at this temperature over a short time span did indeed occur (see Supplementary Figure S4). The explanation for the extreme long-term stability at 90°C may lie in the refolding of previously (partially) inactivated enzyme. This refolding may occur during cooling to room temperature after sample withdrawal and before addition of the *p*NP-arabinoside substrate and the measurement of residual activity. To assess the enzymes’ structural thermostability more directly, the melting temperatures (T_m_) of the enzymes were determined using differential scanning fluorimetry (DSF). The apparent T_m_ values for *Cs*_Gaf159A, *Ch*_Gaf159A, and *Ck_*Gaf159A were 95.5°C, 92.5°C, and 99.0°C respectively (Figure 6), which was consistent with a high intrinsic stability of all three orthologous enzymes.

As for kinetic parameters and product inhibition, the latter was low under presumed physiological conditions, as the enzymes retained more than 50% of their activity in the presence of 500 mM arabinose in the reactions (Supplementary Figure S3). Due to the instability of the substrate *p*NPA at the high assay temperatures (90, 82, and 93°C) used, only short reaction times were applied.

Kinetic parameters with the chromogenic arabinoside substrate *p*NPA were able to be determined only up to a concentration of 10 mM. Results obtained at the standard enzyme concentration of 0.1 µg μl^−1^ (2.65 µM) after 10, 20 and 30 min at the enzymes’ optimal temperature are shown by way of example for *Cs_*Gaf159A at 90°C in Supplementary Figure S4. Substrate saturation was not reached with substrate concentrations up to 10 mM using a 10 min assay. On the other hand, the curves generated after incubation for 20 or 30 min showed some flattening at higher substrate concentrations, which is presumed to be the result of a loss of enzyme activity over time at 90°C (see above). Therefore, the reactions were stopped after 10 min in the following experiments. The curves of substrate concentration vs. reaction rates were recorded for *Cs_*Gaf159A and *Ch_*Gaf159A at various enzyme concentrations. Although the conditions for near saturation of the substrate were not met, the curves suggest a minimum Km for *p*NPA of at least 10 mM.

### Activity of *Cs*_Gaf159A, *Ch*_Gaf159A, and *Ck*_Gaf159A on Arabinose Containing Polysaccharides and AXOS

Overnight incubation of 5 mg ml^−1^ arabinan, arabinoxylan, arabinogalactan, and gum arabic with the three enzymes at different temperatures (65, 70, 75, and 80°C) were analyzed via the DNSA assay. Increased reducing sugar content was measureable only with arabinogalactan (containing arabinose residues linked either α-1,6 to the galactan backbone, or α-1,4 to galactose side chains) when compared to control reactions without enzyme addition. Product analysis by HPAEC-PAD revealed that only arabinose (about 5 mg L^−1^ corrected by enzyme and substrate controls) was liberated after an incubation period of 23 h (Supplementary Figure S5). This only accounts for about 0.6% of the possible release of arabinose, which was calculated to be 0.76 mg ml^−1^. This was calculated assuming 95% purity and 14% arabinose content in larch arabinogalactan (Megazyme data sheet), a substrate concentration of 5 g L^−1^ and the ratio in molecular weight of hydrolyzed to non-hydrolyzed arabinose of 150.13 g mol^−1^: 132.11 g mol^−1^. The calculated specific activity was 0.024 nmol min^−1^ mg^−1^.

**FIGURE 7:**
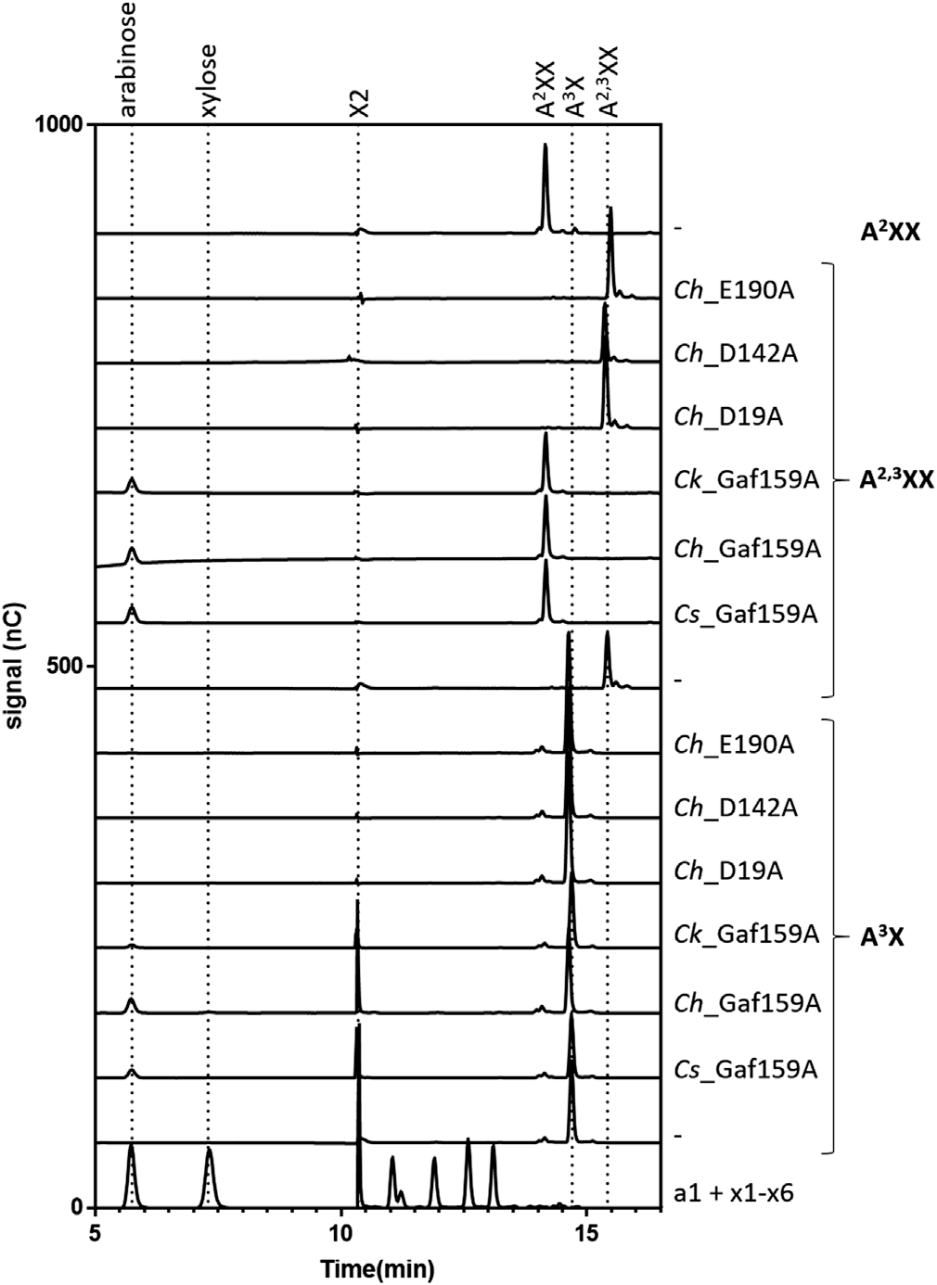
Degradation of A^2,3^XX and A^3^X by *Cs*_Gaf159A, *Ch*_Gaf159A and *Ck*_Gaf159A but not by any of the *Ch*_Mutants. Selected HPAEC-PAD chromatograms of reactions are shown containing 0.1 g L^−1^ substrate (A^3^X, A^2^XX, A^2,3^XX, XA^3^XX, XAXX-Mix or XA^2,3^,XX), 2.4 µM *Cs*_, 0.8 µM *Ch*_, 0.3 µM *Ck*_Gaf159A, or 2.7 µM of the mutants: *Ch*_D19A, *Ch*_D142A, or *Ch*_E190A, 1 x RP, 1 mM CaCl_2_, and were incubated 24 h at 75°C. A complete conversion from A^2,3^XX to A^2^XX, and thus the release of arabinose, was observed for all three wild type enzymes. Small amounts of arabinose and xylobiose (X2) were produced of A^3^X by *Cs-* and *Ch*-, and also in less amounts by *Ck_*Gaf159A. A^2^XX, XA^3^XX, XAXX-Mix and XA^2,3^XX were not used by *Cs*-, *Ch*- or *Ck*_Gaf159A (supplementary Figure S10) Mutants did not show any activity.

In contrast to the lack of activity against polymeric arabinoxylan, all three wildtype enzymes were able to efficiently cleave arabinoxylooligosaccharides (AXOS). HPAEC-PAD analysis (Figure 7; Supplementary Figure S6) revealed that, in 24 h reactions containing 0.1 g L^−1^ substrate, the arabinofuranosidase activity of *Cs*_Gaf159A, *Ch*_Gaf159A, and *Ck_*Gaf159A was able to fully cleave the α-l-1,3-O linkage in A^2,3^XX, yielding A^2^XX and arabinose as the only products. In contrast, in A^3^X-containing reactions under identical conditions only small amounts of arabinose and xylobiose (X2) were released, indicating incomplete hydrolysis of A^3^X.

### Crystallization of *Ch*_Gaf159A and Structure Determination

**FIGURE 8:**
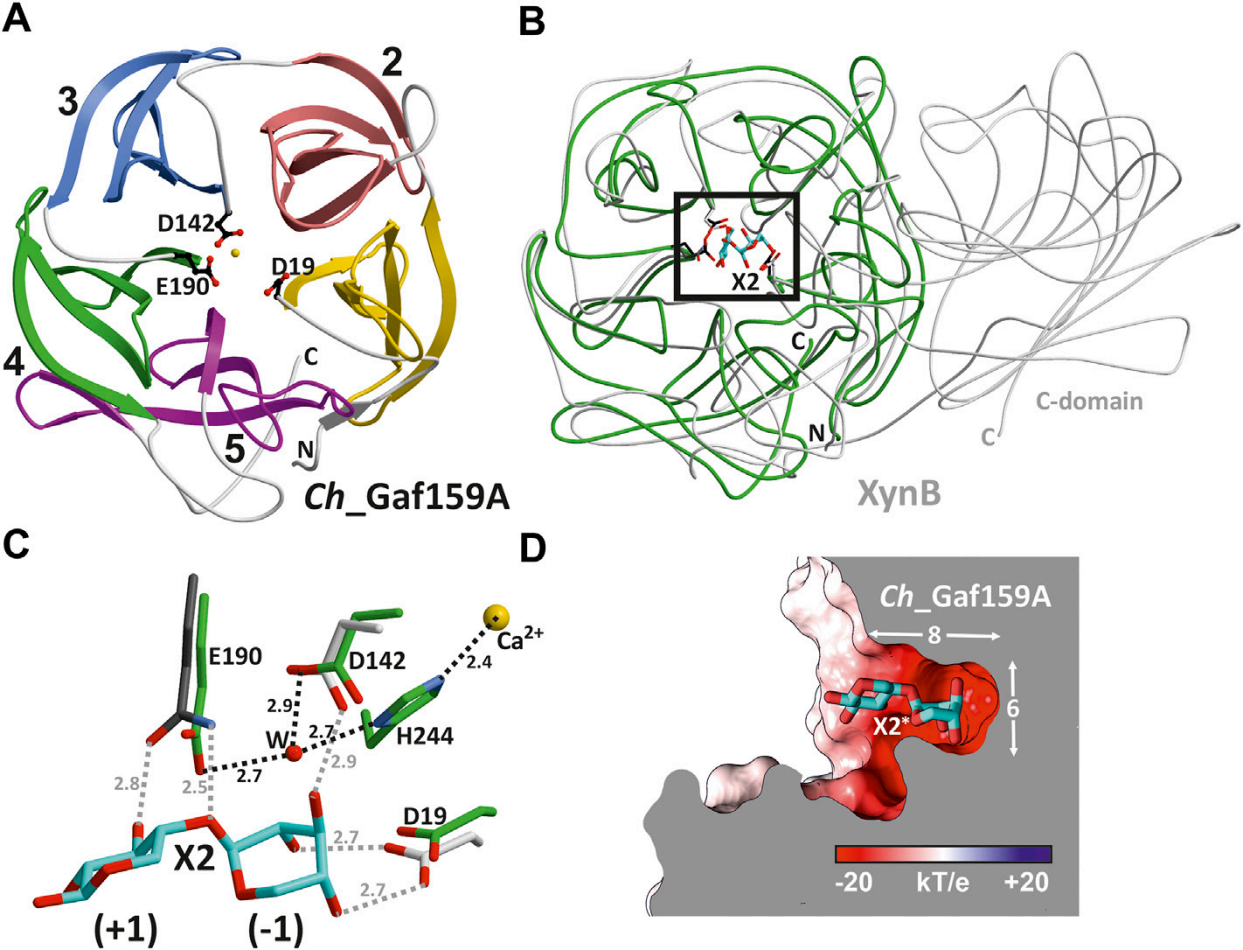
Crystal structure of *Ch*_Gaf159A from *Caldicellulosiruptor hydrothermalis*. **(A)** Monomeric *Ch*_Gaf159A adopts a β-propeller fold (PDB ID 7ZEI) consisting of five blades (ribbon drawing, linker regions in grey, N- and C-terminus marked in capitals) and houses a central metal ion (modelled as Ca^2+^, yellow sphere). The topology corresponds to glycoside hydrolase clan F. Asp19, Asp142 and Glu190 (shown in balls-and-sticks) are conserved residues in this enzyme class and form the catalytic center. **(B)** Superposition of *Ch*_Gaf159A (green) and mutant β-xylosidase (XynB, E186Q, PDB: 5ZQX, grey) in complex with xylobiose (X2, cyan). *Ch*_Gaf159A lacks the C-terminal β-sandwich domain that mediates homodimer formation in XynB (Hong et al., 2018). **(C)** Close up view of the active site (black rectangle in B). In XynB, hydroxy groups of X2 at position (−1) are fixed by an extensive hydrogen bonding network. The introduced E186Q mutation (dark grey) abolishes hydrolytic enzyme activity. Despite low sequence identity (15%) between *Ch*_Gaf159A and XynB, these residues match perfectly (labelled for *Ch*_Gaf159A). Distances are shown in Å, H-bonds for *Ch*_Gaf159A are drawn in black dots. **(D)** Surface representation of *Ch*_Gaf159A colored according to negative and positive electrostatic potentials. The modelled ligand (X2^*^) originates from the structural overlay with XynB and aligns with the specificity pocket (8 Å × 6 Å), indicating exo hydrolase activity of *Ch*_Gaf159A.

*Ch*_Gaf159A was crystallized and its structure determined through molecular replacement to a resolution of 1.7 Å (R_free_ = 15.3%; PDB ID: 7ZEI, see Supplementary Table S3). The stable monomeric state of *Ch*_Gaf159A was confirmed by means of polymerization-induced self-assembly (PISA) (Krissinel and Henrick 2007) calculations and size-exclusion chromatography. The PDB Pisa analysis predicted a solvent-accessible surface area of 13519.7 Å^2^ and −324.2 kcal/mol free energy for folding. The fold of *Ch*_Gaf159A displays striking similarities to that of glycoside hydrolase family 43. A search for homologous topologies using the DALI-server (Holm and Sander 1995) revealed structures with high Z scores but low sequence identities of <20% (structural superposition of representatives with *Ch*_Gaf159A are shown in Supplementary Figure S7). The best hits turned out to be an exo-α-1,5-l-arabinofuranosidase from GH43_26 (root-mean-square deviation (rmsd): 1.5 Å for 173 C_α_ atoms, sequence identity (SI): 17%, Z = 27.0, PDB ID: 3AKI) (Fujimoto et al., 2010) and a mutant derivative (E186Q) of *Bacillus pumilus* β-xylosidase XynB in complex with xylobiose (rmsd 1.7 Å for 181 C_α_ atoms, sequence identity 15%, Z = 26.2, PDB ID: 5ZQX) classified in GH43_11 (Hong et al., 2018). However, *Ch*_Gaf159A is substantially smaller than its homologues, consisting of only a five-bladed β-propeller domain (Figure 8A). The metal ion present in the central cavity was modelled as Ca^2+^ due to the absence of any anomalous signal and the experimental findings for metal ion dependence described above (Figure 8A). Each blade is composed of four mainly antiparallel beta-strands. The overlay with XynB in complex with xylobiose (X2) suggests the substrate binding on the upper part of the barrels center (Figure 8B). The positions of E190, D142, and D19 in *Ch*_Gaf159A exactly matched E186, proposed to be the nucleophilic catalytic base, D127, and D14 in XynB (Figure 8C). The latter two possible catalytic aspartic acid residues of *Ch*_Gaf159A surround the non-reducing end of the substrate in subsite -1 of the binding site. The specific interaction with hydroxides of C2, C3, and C4 in xylobiose determines the orientation of the glycosidic linkage and therefore substrate specificity. E190 in *Ch*_Gaf159A is suggested as being catalytically important given its proximity to the glycosidic O and its description as a catalytic base in XynB. H244 located between D19 and D142, also adopts a putatively important position in coordinating the metal ion (Figure 8C). This matches the findings for CoXyl43 as previously described by Matsuzawa et al. (2017) (Supplementary Figure S8).

Analyses for pronounced specificity pockets at the active site identified a solvent exposed negatively charged ligand binding site with approximately 8 Å in length and 6 Å in diameter (Berka et al., 2012). The surface representation showed two electro negative pockets, one of them capable of incorporating the X2 ligand (Figure 8D). This substrate pocket structurally explains why *Ch*_Gaf159A acts as an exo-arabinofuranosidase, or -galactofuranosidase. The substrate can only enter the pocket at one end and is specifically bound in the -1 subsite (Figure 8D).

The sequence of *Ch*_Gaf159A also structurally aligned (Supplementary Figure S9) with the catalytic residues of GH43_33 HoAraf (PDB 4QQS), an α-l-arabinofuranosidase from *Halothermothrix orenii* (Hassan et al., 2015). The known conserved catalytic nucleophile of HoAraf (E195) aligned with E190 in *Ch_*Gaf159A, the catalytic base D17 (HoAraf) with D19, and D126 in HoAraf corresponded to D142 in *Ch_*Gaf159A. This indicated that all three residues are catalytically important.

### Mutation of Putative Catalytically Essential Amino Acids Results in Loss of Function

**FIGURE 9:**
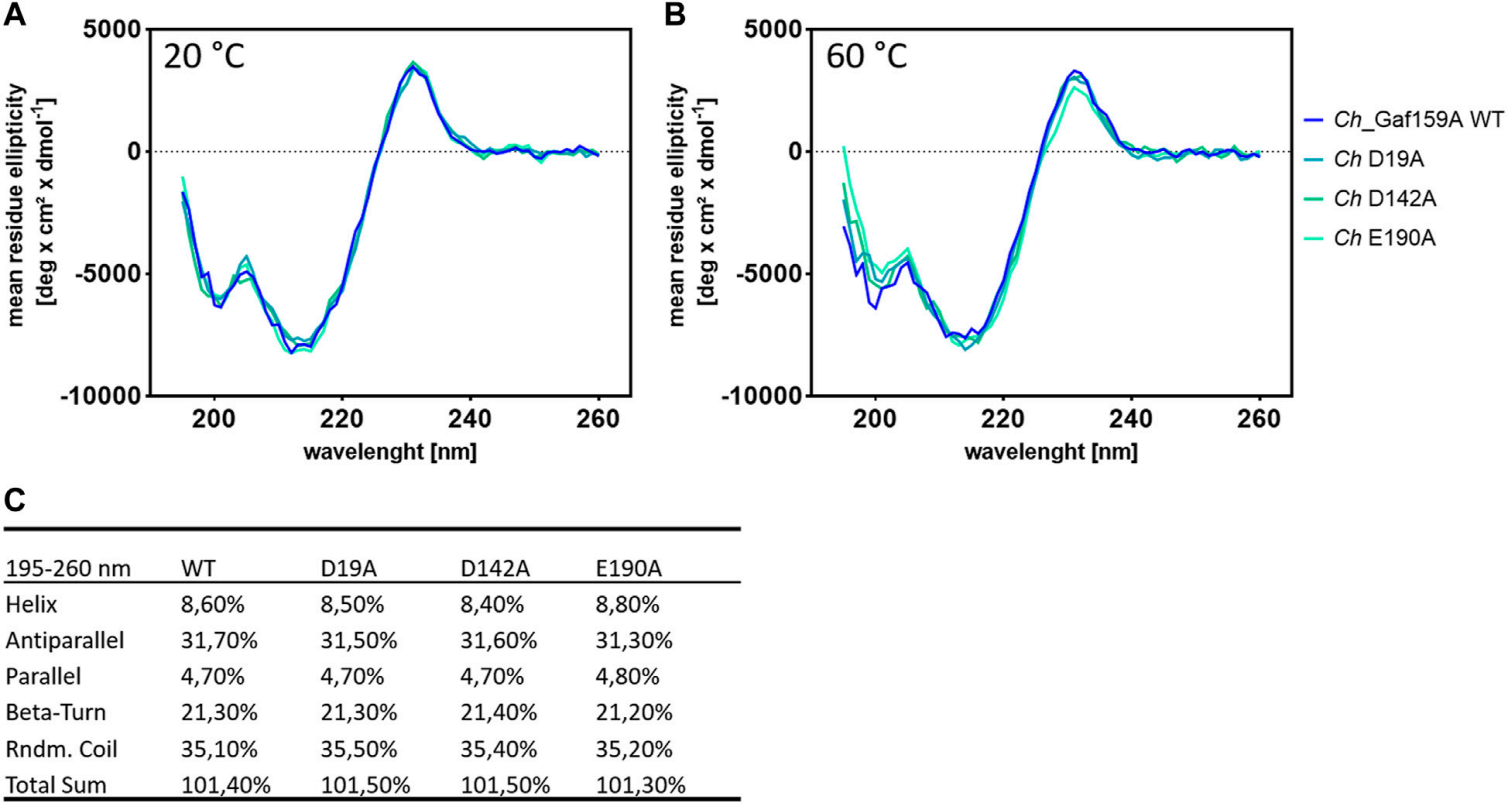
Circular dichroism measurements for *Ch*_Gaf159A and its mutants *Ch*_D19A, *Ch*_D142A and *Ch*_E190A. CD spectra obtained at 20°C **(A)** and 60°C **(B)** and, secondary structure composition revealed by computational spectra analysis at 20°C **(C)**.

Therefore, three variants of *Ch_*Gaf159A were generated at the possible catalytic amino acid position E190, D19 and D142, each containing an alanine residue instead of the proposed catalytic residue. All three mutants lost the ability to cleave *p*NPA, as well as A^2,3^XX and A^3^X (Figure 7), nor did they show any activity against the other tested AXOS (Supplementary Figure S10). The behavior during expression, purification, or protein analysis exhibited change in none of the mutants (Mut I D19A, Mut II D142A, or Mut III E190A) as compared to the wildtype *Ch_*Gaf159A. DSF analyses revealed that the melting temperature T_m_ of Mut II (D142A) was unchanged. In contrast, the T_m_ of Mut I (D19A) was reduced by 7.5°C, and that of Mut III (E190A) increased by 3°C (Figure 6). Since differences in melting temperatures could indicate differences in folding properties, Circular Dichroism (CD) measurements were additionally performed. The far UV CD-spectra of the mutants compared to wild type indicated no changes in secondary structure and probably none in the overall folding properties of the backbone either (Rodger 2018), at 20°C (Figure 9A). In addition, the curves were identical for WT and mutants at 60°C (Figure 9B). Since the wild type still showed about 50% of its activity at 60°C, it can be assumed that the wild type was correctly folded at this temperature, which also applied to the mutants. According to the secondary structure composition deduced from the CD spectra by computational analysis, all *Ch_*Gaf159A variants are composed of 31–32% antiparallel beta-strands, 21% beta turns, and 8–9% helical structures. The proportion of random coils was calculated to be 35–36% in WT and mutants (Figure 9C).

## Discussion

A function-based, experimentally confirmed nomenclature for proteins is needed in order to achieve meaningful annotation of new gene and protein sequences, and to supply references for the functional and structural prediction of proteins (Gupta and Sardar 2021). This work focused on the biochemical and structural characterization of three members of the recently described glycoside hydrolase family GH159 in order to contribute to the overall characterization of this enzyme family. To this end, we chose three GH159 enzymes from the *Caldicellulosiruptor* species *C. saccharolyticus*, *C. hydrothermalis,* and *C. kronotskyensis* and succeeded in obtaining the proteins with a high degree of purity by means of recombinant expression and affinity purification.

In agreement with the first characterized GH159 enzyme, which was a β-d-galactofuranosidase from *Bacteroides cellulosilyticus* WH2 (ALJ59596.1), all three *Caldicellulosiruptor* enzymes showed activity towards the model-substrate *p*NP-β-d-galactofuranoside (*p*NPG). In addition, we observed weaker activity against *p*NP-α-l-arabinofuranoside (*p*NPA). The occurrence of arabinofuranosidase (Abf) and galactofuranosidase (Gaf) activity in one enzyme has also been observed for other enzymes (Tefsen et al., 2012; Pavic et al., 2019). This is plausible because the two sugars differ by only one carbon atom, which is not involved in furanose ring formation (Chlubnová et al., 2010). Due to their β-d-galactofuranosidase activity (EC 3.2.1.146), the gene products of Casc_0437 (ABP66075.1), Calhy_0274 (ADQ06027.1), and Calkro_0290 (ADQ45201.1) were termed β-d-galactofuranosidases (Gafs) according to common nomenclature. However, this study focused on the α-l-arabinofuranosidase activity (EC 3.2.1.55) as this activity had not yet been described for GH159. In addition, the thermostable *Caldicellulosiruptor* enzymes may be useful as added enzymatic activities enhancing the degradation of arabinose-containing lignocellulosic polysaccharides. The complete saccharification of lignocellulose is a desirable goal for the sustainable industrial production of bioethanol and chemicals (Østby et al., 2020), while galactofuranose-containing substrates are less common in lignocellulosic biomass (Seiboth and Metz 2011).

In accordance with the high sequence identity between the three enzymes *Cs_*Gaf159A, *Ch_*Gaf159A and *Ck*_Gaf159A (more than 90% with 100% query coverage), their biochemical characteristics in terms of activity, substrate specificity, pH optimum (between 5.5 and 6, measured at 80 °C) were identical. Additionally, cation dependence, and to some extent also their temperature preference for activity (maximum activity in a 20 min assay between 82 and 93°C), as well as thermal stability, were all similar. Like some other glycoside hydrolases (Ahmed et al., 2013; Baudrexl et al., 2019), *Cs*_Gaf159A, *Ch*_Gaf159A, and *Ck_*Gaf159A are activated by divalent metal ions. The greatest effect was observed with either MnCl_2_ or CaCl_2_ ions (Figure 3).

As can be expected for proteins of extremely thermophilic bacteria that grow optimally at temperatures of 70°C, the *Caldicellulosiruptor* enzymes *Cs*_Gaf159A, *Ch*_Gaf159A, and *Ck*_Gaf159A displayed a high level of resistance against thermal inactivation. In particular, *Cs*_Gaf159A was able to be incubated for 24 h at its temperature of maximum activity of 90°C, apparently without losing activity (Supplementary Figure S3). On the other hand, an indication that heat inactivation of *Cs*_Gaf159A at this temperature did occur was obtained by varying the assay time between 10 and 30 min (see Supplementary Figure S4). The explanation for the apparent extreme long-term stability at 90°C may lie in the efficient refolding of previously (partially) inactivated enzyme enabled by the assay conditions. In this case, after incubation of *Cs*_Gaf159A at 90°C and sample withdrawal, the samples were cooled to room temperature before the *p*NP-arabinoside substrate was added for measurement of residual activity. During this cooling period, refolding of previously (partially) inactivated enzyme may have occurred, meaning that the reversibility of potential temperature-induced (partial) unfolding rather than the thermostability had been tested. If this hypothesis, which has not been tested within this study, is correct, then *Cs*_Gaf159A is the most flexible protein of this study. Namely, it was probably able to correctly refold after incubation at 90°C for 24 h and regain its original activity. This is an often observed phenomenon with thermophlic proteins and for instance has been reported for the catalytic domain of xylanase Xyn10B (Araki et al., 2006). As an outlook, it would be interesting to study the folding and unfolding properties of *Cs*_Gaf159A in particular.

Temperature-dependent denaturation was evaluated by determining the enzymes’ melting temperatures (T_m_) *via* DSF. The T_m_ reflects a protein’s intrinsic thermal stability, which is usually higher than the physiological temperature to which the protein is exposed to in its natural environment (Menzen 2014). With this approach, *Ck*_Gaf159A showed the highest T_m_ (99°C), which is consistent with the finding that it had the highest “optimal” temperature, i.e., the temperature for maximum activity in a 20 min assay (T_Opt_ = 93°C), followed by *Cs*_Gaf159A (T_m_ = 95.5°C) and *Ch*_Gaf159A (T_m_ = 92.5°C). Compared to the 20 min optimal temperatures the melting temperatures of *Cs*_Gaf159A, *Ch*_Gaf159A and *Ck*_Gaf159A were 5.5°C, 10.5°C, and 6°C higher, respectively. The melting curves indicate that the enzymes start to partially unfold at their “optimum” temperatures (90°C, 82°C, and 93°C), which may account for the observed activity loss over time. The dye used for DSF, SyproOrange, binds to hydrophobic regions in proteins. Considering the high overall sequence similarity of the enzymes and the fact that the number of hydrophobic residues was almost the same, the differences in both T_m_ (92.5—99°C) and T_Opt (20min)_ values (82—93°C) for the investigated enzymes were relatively high. The counts of hydrophobic residues were 23, 24, and 24 for *Cs*_Gaf159A, *Ch*_Gaf159A, and *Ck*_Gaf159A, respectively (Supplementary Table S4). The melting curves suggest that all of the enzymes investigated could be stable at approximately 70°C for extended periods of time, which nicely matches the optimal growth temperature of *Caldicellulosiruptor* species (see above). The T_m_ values were much higher than the optimum growth temperature and a significant resistance against thermo-inactivation has been observed, even at temperatures 20°C above the optimum growth temperature. This may be advantageous for bacteria thriving in hot springs, because significant temperature fluctuations often found in such habitats would not lead to irreversible enzyme inactivation. With regard to the methodology used for assessing the thermostability of enzymes, DSF clearly is a more suitable method than activity-based methods. In activity-based methods, incubation times as well as refolding of previously unfolded, and thereby inactivated, enzyme play a crucial role. Adding preheated substrate to the preincubated enzymes without cooling of the enzymes would avoid refolding during the cooling period, but this approach is experimentally challenging at temperatures around and above 80 °C.

Due to the low specificity for *p*NPA and the instability of *p*NPA at higher temperatures, kinetic parameters were not able to be evaluated. However, using 10 mM as the highest *p*NPA concentration and enzyme concentrations of 0.036 µM it can be concluded that the Michaelis Menten constant K_m_ must at least be higher than the substrate concentration of 10 mM (Li 2017). This and the values obtained using *p*NPG as a substrate (4.2–8.5 mM), were higher than most of the reported K_m_ values for Abf, which range from about 0.1 to 1.5 mM *p*NPA (Inacio et al., 2008; Matsuzawa et al., 2017; Coconi Linares et al., 2022).

The substrate specificity of the enzymes was tested using a range of glycosidic substrates. *Cs*_Gaf159A, *Ch*_Gaf159A and *Ck_*Gaf159A were not able to cleave the backbone or the sidechain arabinosidic linkages (arabinose residues linked α-1,3 and α-1,5 to backbone arabinoses) present in arabinan, arabinoxylan (arabinose residues linked α-1,2 and/or α-1,3 to xylan backbone), or gum arabic. The latter is a complex heteropolysaccharide containing l-arabinose (Pragnesh and Ankur 2018) in the main and side chains (Musa et al., 2018) and probably at least some non-reducing terminal Ara*f*-residues (Tagawa and Kaji 1969). In contrast, the enzymes exhibited arabinosidase activity on the polysaccharide arabinogalactan, which contains sidechains with terminal arabinofuranose residues (Robinson et al., 2008) which may be cleaved off. The absence of the enzymes’ activity with arabinan, arabinoxylan and gum arabic, as well as the limited activity on arabinogalactan in addition to their activity on small substrates such as *p*NPA and various AXOS indicates that the arabinosidase activities of *Cs*_Gaf159A, *Ch*_Gaf159A, and *Ck_*Gaf159A should be affiliated with the type A arabinofuranosidases (Bourgois et al., 2007). Furthermore, the lack of signal peptides in all three enzymes indicates their intracellular localization. This matches their preference for low molecular mass substrates such as arabinose-containing oligosaccharides, which are far more likely to be encountered intracellularly than polysaccharides are (Reintjes et al., 2017).

In addition to preferences for poly- or oligosaccharides, or for substrates of a small size, further specificity characteristics have been described for a special group of arabinofuranosidases, the arabinoxylan arabinofuranohydrolases (Axh). A simple nomenclature was proposed by Vandermarliere et al., 2009 for different specificities, e.g. Axh-d3 (van Laere et al., 1999; van den Broek et al., 2005; Sørensen et al., 2006), Axh-m2,3 (van Laere et al., 1999; Wang et al., 2014), Axh-m3 (Kormelink et al., 1993) or Axh-m,d2 (Cartmell et al., 2011). This nomenclature depends on whether arabinofuranose residues are cleaved from double substituted xylose residues (d), or from mono-substituted xyloses (m) and taking into account the preference for 1,3- or 1,2-linkages. Adapting this nomenclature to the arabinofuranosidase activities of the enzymes of this study against AXOS, Axh-m,d3 activity seems to be insufficiently specific since *Cs*_Gaf159A, *Ch*_Gaf159A, and *Ck_*Gaf159A preferentially cleave α-1,3 linkages only when the non-reducing end of the arabinosylated xylose residue is free, such as in A^2,3^XX and also in A^3^X. In contrast, XA^3^XX, or XA^2,3^XX, are not substrates.

Different preferences for internal and external substituents have previously been described, e.g., a GH51 arabinofuranosidase with Axh-m,d activity on the terminal, but Axh-m activity on internal Xylp residues (Koutaniemi and Tenkanen 2016). The fact that the *Caldicellulosiruptor* enzymes cleaved only small amounts of arabinose from A^3^X, and that polymeric substrates were not efficiently hydrolyzed, indicates either that only oligosaccharides of a certain size can be fitted into the substrate-binding pocket of the enzyme, or that double-substituted xylose residues are preferred. To verify this experimentally, it would be helpful to test additional substrates with substitutions (single or double) at the first xylose residue (counted from the non-reducing end), such as A^3^XX, A^3^XXX, A^2,3^X, and A^2,3^XXX. However, these are not commercially available. Even though the actual role of the enzymes *Cs*_Gaf159A, *Ch_Gaf159A,* and *Ck*_Gaf159A for the producing bacteria has never been investigated, a role in the intracellular degradation and further utilization of arabinose-containing oligosaccharides in concert with backbone cleaving enzymes would be conceivable, as described for AbfA and Abf2 of *B. subtilis* (Inacio et al., 2008).

Crystallization and elucidation of the molecular structure of the WT *Ch*_Gaf159A (PDB: 7ZEI) protein revealed a 5-fold beta propeller structure, which is a conserved fold for glycoside hydrolases present in clan GH-F (GH43, GH62 and GH117) and GH-J (GH32 and GH68). In contrast to clan GH-J enzymes, which are active on fructose-containing substrates and retain the net configuration, GH-F enzymes invert the configuration at the anomeric carbon of their β-d-xylopyranosylated, α-l-arabinofuranosylated, or β-d-galactofuranosylated substrates. The specificity towards these three sugar moieties is conceivable, as the orientation of the ring substituents is identical for C1, C2, C3 and C4 (Supplementary Figure S11). The cleavage specificity of *Cs_*Gaf159A, *Ch_*Gaf159A and *Ck*_Gaf159A for β-d-Gal*f* and α-l-Ara*f* substituted substrates, as well as the similar positioning of the catalytic residues compared with GH43_11 XynB, suggests that also GH159 enzymes act *via* an inverting cleavage mechanism and should be included in GH clan F. However, the catalytic mode was not investigated so far. The central ion which was modelled in the *Ch_*Gaf159A structure as Ca^2+^ due to the experimental findings described before matches the results obtained for other GH-F enzymes, such as GH62 4080 (Wang et al., 2014), GH117 4AK7 (Hehemann et al., 2012), or GH43_1 5GLR (Matsuzawa et al., 2017), whose structures also contained a metal ion.

Residues E190, D142, and D19, which in the three-dimensional *Ch_*Gaf159A structure are found in close proximity to each other in the center of the channel formed by the β-propeller, perfectly matched the catalytically essential amino acid residues of a structurally similar enzyme of GH43_11 (XynB; 5ZQX; SI: 15%) in an overlay of both enzyme structures and also in a structural alignment with HoAraf (4QQS; SI: 27%). Therefore, they were suggested as possible catalytical residues for *Ch*_Gaf159A. The complete loss of activity of the *Ch_*Gaf159A mutants D19A, D142A and E190A, which was tested with *p*NPA, AXOS and arabinogalactan, confirmed this prediction. The topology of the crater-shaped substrate specificity pocket also revealed the structural basis for the exo-activity that has been experimentally observed. In analogy, the structural homolog 5ZQX was reported to display β-xylosidase activity but had no activity toward the polysaccharide xylan (Xu et al., 1991; Hong et al., 2018). The bifurcated binding pocket reinforces the experimentally obtained assumption that d-AXOS are preferred over m-AXOS. However, the crystallization of the glutamine mutant E190Q in complex with the substrate A^2,3^XX to confirm the specific binding of d-AXOS was not successful yet. The liganded structure would have been necessary to shed light on the orientation of the substrate backbone and the residues involved, which would have allowed the analysis of the observed preference towards low molecular mass substrates.

In conclusion, this study elucidated the first 3D structure of a representative of the glycoside hydrolase family GH159. The analyses revealed molecular details of substrate binding and the catalytic mechanism of enzymes belonging to this family. α-l-arabinofuranosidase activity (EC 3.2.1.55), which has not been reported for the most closely related GH159 enzyme (BcellWH2_02356; Helbert et al., 2019) was biochemically characterized for the three GH159 members of this study, in addition to β-d-galactofuranosidase activity (EC 3.2.1.146). In retrospect, the observation of arabinofuranosidase activity in GH159 is expected due to structural similarities with GH43 family members. Taken together, the work reported here adds a new cleavage specificity not known before to glycoside hydrolase family GH159 as well as its protein fold.

## Materials and Methods

### Selection of Enzymes and Homology Analysis

The Carbohydrate Active Enzymes (CAZy) database (http://www.cazy.org/Glycoside-Hydrolases.html; Lombard et al., 2014) was scoured, and homology analysis was performed using the NCBI tool BLASTp (https://blast.ncbi.nlm.nih.gov/Blast.cgi). Signal peptides were predicted using the SignalP 4.1 server (http://www.cbs.dtu.dk/services/SignalP-4.1/; (Petersen et al., 2011). Structural information was extracted from the RCSB PDB Protein Data Bank (http://www.rcsb.org/). Structural alignments were created using HHpred, a MPI Bioinformatics Toolkit (https://toolkit.tuebingen.mpg.de/tools/hhpred; Zimmermann et al., 2018) to compare conserved catalytic amino acids.

### Anaerobic Cultivation of *Caldicellulosiruptor* Strains and Isolation of Genomic DNA

The bacterial strains *C. saccharolyticus* DSM 8903, *C. hydrothermalis* 108 (DSMZ 18901), *C. kronotskyensis* 2002 (DSMZ No. 18902) were obtained from the DSMZ (Braunschweig, Germany). Dried cells were rehydrated with 500 µl GS2 medium (Johnson et al., 1981; Koeck et al., 2015) for 30 min under anaerobic atmosphere in an anaerobic chamber (98% N_2_, 2% H_2_; Coy Laboratory Products). An aliquot of 250 µl was used to inoculate 50 ml GS2 medium in butyl rubber-stoppered serum bottles with 0.1% cellobiose. Another 100 µl were plated in the anaerobic chamber onto a GS2 agar plate (1.8% agar). Cells were cultivated anaerobically at 65°C for 5 days (bottles without shaking). Genomic DNA of a clonal culture inoculated from a single colony was isolated using the Gentra® Puregene® kit for Yeast and Bacteria Kit (Qiagen). Briefly, 5 ml of the bacterial culture was centrifuged for 1 min at 13,000 rpm. After discarding the supernatant, the protocol for DNA isolation was followed in accordance with the manufacturer’s description for Gram-positive bacteria. However, 1.5 μl of lysozyme (730 U ml^−1^) was used instead of 1.5 μl of the Lytic Enzyme Solution. PCR-amplified DNA fragments were purified using NucleoSpin® Gel and PCR Clean-up kits (Macherey-Nagel).

### Cloning, Expression, and Purification of Recombinant Enzymes

Recombinant *Escherichia coli* strains DH10B (Thermo Fisher Scientific, Waltham, MA, United States) and BL21 Star (Invitrogen, Carlsbad, CA, United States) were aerobically grown in LB-Lennox medium with 50 µg ml^−1^ kanamycin at 180 rpm and 37°C. Plasmids were isolated from overnight cultures using the NucleoSpin Plasmid kit from Macherey-Nagel (Düren, Germany). Amplified DNA was purified using the NucleoSpin® Gel and PCR Clean-up (Macherey-Nagel, Düren, Germany). Plasmid pET24c (+) (Merck, Darmstadt, Germany) was amplified in *E. coli* DH10B, cut with NdeI and XhoI (New England Biolabs GmbH, Frankfurt, Germany) and purified from an 1% agarose gel. The genes coding for the GH159 enzymes with arabinofuranosidase activity from *C. saccharolyticus* (*Cs_*Gaf159A; ABP66075.1), *C. hydrothermalis* (*Ch_Gaf159A*; ADQ06027.1) and *C. kronotskyensis* (*Ck_*Gaf159A; ADQ45201.1) were amplified from genomic DNA by PCR using Phusion High-Fidelity DNA Polymerase (Thermo Fisher Scientific, A, United States), genomic DNA of the respective strain and primer pairs as listed in Table 1. Cloning of pET24c(+) constructs was accomplished by Gibson Assembly (Gibson et al., 2009) using purified PCR product and restricted vector as described above, followed by transformation of strain *E. coli* DH10B. Sanger sequencing at Eurofins Genomics (Ebersberg, Germany) verified sequence integrity before enzyme production in *E. coli* BL21 Star™, isolation and purification by immobilized metal affinity chromatography (IMAC) was carried out as described previously (Baudrexl et al., 2019). The eluates from IMAC were incubated at 50°C for 15 min to denature remaining *E. coli* proteins followed by precipitate removal by centrifugation. Size and purity of the proteins were confirmed by SDS-PAGE (Laemmli 1970). Protein concentration was determined spectrophotometrically at 280 nm under denaturing conditions (5 M urea) using the molar extinction coefficients, calculated by the ExPaSy ProtParam tool (https://web.expasy.org/protparam/) from the Swiss Institute of Bioinformatics (Artimo et al., 2012).

**TABLE 1:** Primers for amplification of gene fragments for Gibson-Assembly and site directed mutagenesis.

Name	5′-3′-Sequence	Target to be amplified
fw_Cs	CTT​TAA​GAA​GGA​GAT​ATA​CAG​TGC​CAA​AGC​CAC​CA	Csac_0437
rev_Cs	CAG​TGG​TGG​TGG​TGG​TGG​TGC​GCA​AAC​AAA​TTA​GGA​AGA​C	Csac_0437
fw_Ch_Ck	CTT​TAA​GAA​GGA​GAT​ATA​CAG​TGC​CAA​GAC​CAC​CA	Calhy_0274, Calkro_0290
rev_Ch	CAG​TGG​TGG​TGG​TGG​TGG​TGC​GCA​AAT​AAA​TCC​GGA​AGA​TAA​A	Calhy_0274
rev_Ck	CAG​TGG​TGG​TGG​TGG​TGG​TGC​GCA​AAT​AGA​TCC​GGA​AGA​TA	Calkro_0290
fw_Ch_D19A	CGGCAG**C**CCCCACCATCA	pET-ChGaf-D19A
rev_Ch_D19A	TGATGGTGGGG**G**CTGCCG	pET-ChGaf-D19A
fw_Ch_D142A	GTT​ATC​G**C**TGC​ATG​CGT​TTT​C	pET-ChGaf-D142A
rev_Ch_D142A	GAA​AAC​GCA​TGC​A**G**CGA​TAA​C	pET-ChGaf-D142A
fw_Ch_E190A	TCA​AG**C**GGG​GCC​AAA​CGT​C	pET-ChGaf-E190A
rev_Ch_E190A	GAC​GTT​TGG​CCC​C**G**CTT​GA	pET-ChGaf-E190A

Non-matching nucleotides for introduction of mutations are bolded.

### Crystallization and Structure Determination

Crystals of *Ch*_Gaf159A (Genbank: ADQ06027.1, Uniprot: E4QB49) from *C. hydrothermalis* were grown at 20°C within 14 days to a final size of 300 μm × 100 μm × 100 μm by using the sitting drop vapour diffusion method. The drops contained 0.3 µl protein (30 mg ml^−1^) and 0.1 µl reservoir (100 mM 2-(N-morpholino)-ethanesulfonic acid, pH 6.5, 30% (w/v) polyethylene glycol 8000, and 5% (v/v) glycerol). Before exposure to X-rays, crystals were soaked in a mixture of mother liquor and glycerol (5:1, v/v) for 30 s and vitrified in liquid nitrogen. A native diffraction data set was collected using synchrotron radiation at the X06SA-beamline, SLS, Switzerland. Reflection intensities were evaluated with the program package XDS and data reduction was carried out with XSCALE (Kabsch 2010) (Supplementary Table S3). Initial phases were obtained by Patterson search calculations with PHASER (McCoy et al., 2007) using a starting model predicted by alphafold2 (Jumper et al., 2021). Six monomers could be positioned in the asymmetric unit. Phases were improved by cyclic non-crystallographic averaging methods, resulting in an electron density map that allowed to correct misaligned secondary structure elements and loop connections. The structure was completed in successive rounds of model building with COOT (Emsley and Cowtan 2004) and refined with REFMAC5 (Vagin et al., 2004). Water molecules were placed with ARP/wARP solvent (Perrakis et al., 1997). The determined crystal structure has superb crystallographic values (Rcrys = 0.128 and Rfree = 0.153), was proven to fulfil the Ramachandran plot (Supplementary Table S3) and evaluated by MolProbity (Williams et al., 2018). Interface areas were calculated with PISA (‘Protein interfaces, surfaces and assemblies’ service at the European Bioinformatics Institute) (Krissinel and Henrick 2005). Coordinates and structure factor amplitudes have been deposited in the RCSB Protein Data Bank under the accession code 7ZEI.

### Mutant Construction by Site Directed Mutagenesis

Mutations were introduced into the gene sequences *via* site directed mutagenesis. For this, linear fragments with overlapping ends were amplified using PCR reactions with reverse complementary primer pairs with single nucleotide changes as indicated in Table 1 and the constructed vector pET24c-*Ch*Gaf159A as template. Reactions were subjected to complete digestion with DpnI to remove non-mutated methylated template DNA isolated from the cloning strain *E. coli* DH10b (Marinus and Løbner-Olesen 2014). Transformation of this strain using the resulting linear DNA with overlapping ends lead to self-circularization upon replication in the transformed cells. The expected mutations were verified by sequencing.

### Activity Assays With *Para*-Nitrophenyl Glycosides for Characterization of Biochemical Properties

Standard reactions with *p*NP-glycosides, purchased from Megazyme (Wicklow, Ireland), were performed in a total volume of 50 µl with 1 mM substrate and 2.65 µM enzyme (1 µg μl^−1^) in standard reaction buffer (100 mM MOPS pH 6.5, 50 mM NaCl, 10 mM CaCl_2_) for 20 min at the indicated temperature. For a first screening, *p*NP-glycoside reactions were incubated at 65°C, the optimal growth temperature of *Caldicellulosiruptor*. For further biochemical characterization, the respective apparent “temperature optima” (temperatures at which maximum activity was measured under standard assay conditions in a 20 min assay) were used, i.e., 90°C for *Cs_*Gaf159A, 82°C for *Ch_*Gaf159A, and 93°C for *Ck_*Gaf159A. Reactions were performed in triplicates and stopped by adding 100 µl 1 M Na_2_CO_3_. Color formation from released *p*NP was quantified by spectrophotometric measurement of 100 μl at 405 nm and calculated using a standard curve created with *p*NP as reference.

Activity on various *p*NP glycosides was assessed by prolonged overnight incubation to detect even very low activity. Dependency on and response to ions was examined by using 100 mM MOPS buffer pH 6.5 in standard reactions with and without addition of salt solutions (1–10 mM of CaCl_2_, NaCl, CuCl_2_, NiSO_4_, KCl, MnCl_2_, MgCl_2_, FeCl_3_, ZnCl_2_, CoCl_2_, NH_4_Cl, K_2_HPO_4_, KSO_4_, KNO_3_, or K_2_CO_3_). For this purpose, the storage buffer for the enzymes was changed to 0.1 M MOPS pH 6.5 without additional ions, either by diafiltration using Vivaspin columns, cutoff 10 kDa (Sartorius AG, Goettingen, Germany), or by using PD-10 desalting columns (Cytiva Europe GmbH, Freiburg, Germany). Temperature optima were determined by measuring activities in a gradient thermocycler (TAdvanced 96S, Biometra, Göttingen, Germany), with a span of 40°C over 12 reactions.

Because the pH tolerance strongly depends on the temperature, an established high throughput method was used to analyze the pH optima as described by Herlet et al. (2017). Citric acid—phosphate buffer was prepared in the style of (McIlvaine 1921), but stock solutions were supplemented with 0.1 M NaCl (McIlvaine+) as described by Herlet et al. (2017). This buffer system (pH 4.5–8) was used in a ratio of 1:2, whereas MOPS buffer (pH 6–7.4) was used in final concentrations as in the standard reactions (0.1 M). Buffers were prepared at room temperature. To take the temperature dependency into account, pH values were additionally measured at 80 °C to use actual pH values under reaction conditions to visualize the pH optima. Values for both buffers at RT and 80°C can be found in Supplementary Table S2.

### Kinetic Analysis

Kinetic parameters v_max_ and K_m_ as known from the Michaelis Menten equation $\left(v_{0}=\frac{v_{\max}\times \left[S\right]}{K_{m}\left[S\right]}\right)$ were calculated from substrate concentration vs. reaction velocity graphs. Standard reactions were performed with final substrate concentration up to 20 mM and enzyme concentrations were reduced to 0.001 µg μl^−1^ (equal to 0.027 µM *Cs_*Gaf159A). The reaction time was reduced to 10 min to minimize the effect of decrease in specific activity with time.

### Thermostability

For activity based stability tests standard reactions were preincubated at different temperatures and then briefly cooled on ice before the substrate was added. To assess intrinsic protein thermal stability, DSF was performed in a C1000 Touch Thermal Cycler equipped with the CFX96 Real-Time System (Bio-Rad). Reactions contained 4 µM protein in reaction buffer (100 mM MOPS pH 6.5, 10 mM CaCl_2_, 50 mM NaCl) and 20x SYPRO® Orange Protein Gel Stain (Merck KGaA, Darmstadt, Germany) diluted in the respective buffer in a total volume of 50 µl. Triplicates were pipetted on ice in white RT-PCR 96-well plates, sealed with Microseal®B Adhesive Sealer (Bio-Rad, MSB-1001). Samples were incubated at the initial temperature (25°C, or 50°C) for 10 min before the temperature was increased every 15 s by 1°C each. Fluorescence was measured each time before the temperature changed. The measured values and respective temperatures were recorded at the respective time points. Interpretation of the saturation curves was performed using the xy analysis method “Smooth differentiate curve” in Graphpad Prism 7.00.

### Secondary Structure Comparison by Circular Dichroism Spectroscopy

CD spectroscopy was conducted with 0.1 µg μl^−1^ enzyme in 10 mM Tris pH 6.5, using a Jasco–J710 CD spectrometer (Oklahoma City, OK, United States), a 1 mm cuvette, a range from 195 to 260 nm, and the signal average of 10 scans at 20 and 60°C as described by Stelzer et al. (2008). Secondary structure compositions were obtained after calculation of molar elipticity from signals in mdeg on the basis of reference spectra (Poschner et al., 2007).

### Activity on Arabinose Containing Polysaccharides and Arabinoxylan-Derived Oligosaccharides

The reactions used to test the ability of the enzymes to cleave arabinose from polysaccharides contained 0.5% wheat medium viscosity arabinoxylan (P-WAXYM, Megazyme, Wicklow, Ireland), sugar beet arabinan (P-ARAB, Megazyme), larch arabinogalactan (P-ARGAL, Megazyme) or acacia gum arabic (Merck, Darmstadt, Germany), 1x RP (100 mM MOPS pH 6.5, 50 mM NaCl, 10 mM CaCl_2_), and 2.65 µM of the respective enzyme. Reactions were incubated overnight at 65, 70, 75, or 80°C. Preferences for specific arabinosyl linkages in arabinoxylan-derived oligosaccharides (AXOS) purchased from Megazyme were tested using an 24 h incubation at 75°C and just 0.01% (100 mg L^−1^) of the respective substrate as follows: Either 3^2^-α-l-arabinofuranosyl-xylobiose (A^3^X), 2^3^-α-l-arabinofuranosyl-xylotriose (A^2^XX), 2^3^,3^3^-di-α-l-arabinofuranosyl-xylotriose (A^2,3^XX), 2^3^,3^3^-di-α-l-arabinofuranosyl-xylotetraose (XA^2,3^XX), 3^3^-α-l-arabinofuranosyl-xylotetraose (XA^3^XX), or a mixture of XA^3^XX and 2^3^-α-l-arabinofuranosyl-xylotetraose (XA^2^XX) which is referred to as XAXX-Mix. The number in the exponent indicates which xylose counting from the reducing end, and the integer of the base shows which oxygen atom of this xylose is substituted with arabinose (Supplementary Figure S12; abbreviations adopted from Fauré et al., 2009).

### Analysis of Hydrolysates by Means of DNSA Assay and HPAEC-PAD

Hydrolysates were analyzed for the liberated reducing ends using the dinitrosalicylic acid method (DNSA), as described elsewhere (Wood and Bhat 1988). To summarize, 50 µl of reactions, or arabinose standard solutions (0.2–2 mg ml^−1^) were mixed with 75 µl reagent (10 g L^−1^ dinitrosalicylic acid, 200 g L^−1^ potassium sodium tartrate, 10 g L^−1^ NaOH, 0.5 g L^−1^ Na_2_SO_4_, 2 g L^−1^ phenol) and heated at 95°C for 10 min. After measuring 100 μl at 540 nm, values were interpolated from arabinose standard curves using non-linear regression in GraphPad Prism 7.

High performance anion exchange chromatography with pulsed amperometric detection (HPAEC-PAD) of polysaccharide and oligosaccharide hydrolysates was conducted as described (Mechelke et al., 2017; Baudrexl et al., 2019) at an adjusted gradient: During the first 10 min, a gradient from 0 to 100 mM sodium acetate was used; in the following 20 min a second linear gradient to a final concentration of 800 mM NaOAc was suitable for separating the arabinoxylan-derived oligosaccharides in a shorter time. In addition, the arabinose was quantified using arabinose solutions (12.5–200 mg L^−1^) as external standards.

## Data Availability

The datasets presented in this study can be found in online repositories. The names of the repository/repositories and accession number(s) can be found in the article/Supplementary Material.
