# Supplementary material for: Biochemical and Structural Characterization of Thermostable GH159 Glycoside Hydrolases Exhibiting α-L-Arabinofuranosidase Activity
Source: Front Mol Biosci. 2022 Jun 29;9:907439. doi: 10.3389/fmolb.2022.907439 (PMC9278983; doi:10.3389/fmolb.2022.907439)
Supplement: Supplementary file 1 [file DataSheet1.PDF]

Supplementary Material

1 Supplementary Figures

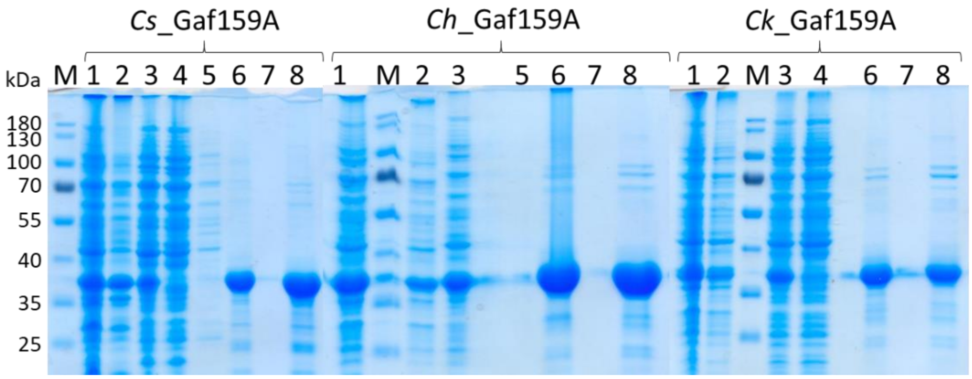

Figure S 1. SDS-PAGE analysis of protein purification steps. Samples were mixed with 4 x SDS-loading buffer (0.25 M Tris, 40% w/v glycerol, 0.29 M SDS, 0,57 β-mercaptoethanol, 0.02% bromophenol blue, HCl to pH 6.8), for samples 1–4 5 µL were applied in each case, for samples 5–7 10 µl each. M: PageRuler™ Prestained Protein Ladder, 10 to 180 kDa, 1: cell lysate, 2: resuspended pellet, 3: cell extract, 4: flowthrough FPLC, 5: FPLC wash, 6: pooled eluates, 7: precipitate after heat treatment (15 min, 50 °C), 8: soluble protein after heat treatment

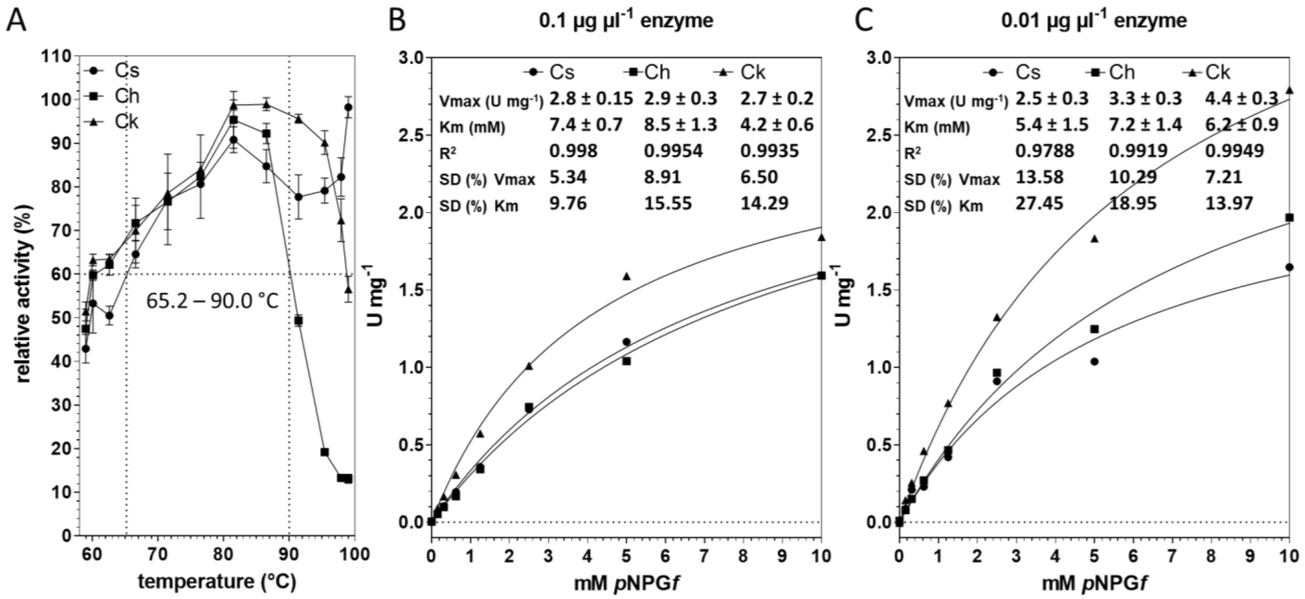

Figure S 2. Galactofuranosidase activity using pNPG as substrate. For the determination of the temperatures resulting in maximal activity of the Caldicellulosiruptor enzymes Cs_Gaf159A, Ch_Gaf159A and Ck_Gaf159A, (A) reactions with 0.1 µg µL^{-1} were incubated at various temperatures for 20 min (n=3), whereas kinetic parameters were analysed after an incubation for only 10 min with either 0.1 µg µl^{-1} (B) or 0.01 µg µl^{-1} (C) enzyme and curves were analysed in Graphpad using Michaelis Menten equation (n=1). All values and standard deviations (SD) were calculated by GraphPad Prism.

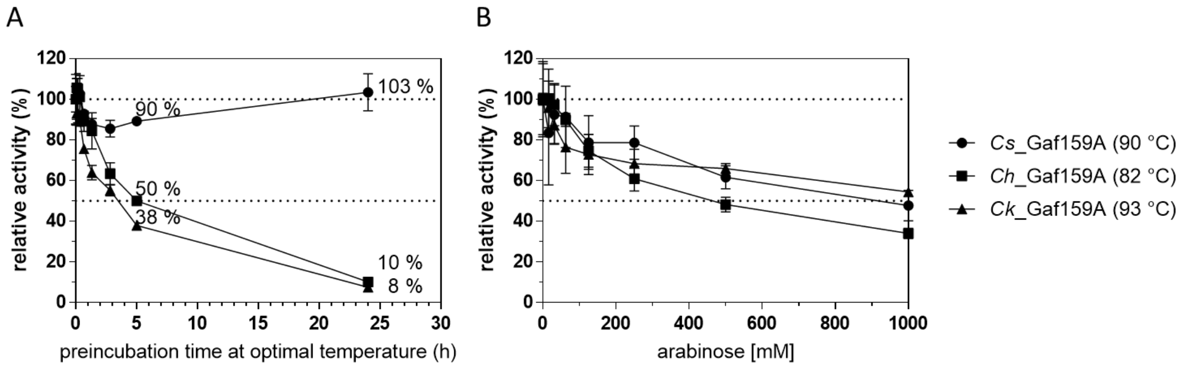

Figure S 3. Residual arabinofuranosidase activities of Cs_Gaf159A, Ch_Gaf159A and Ck_Gaf159A after preincubation at the enzymes optimal temperatures (A) or in presence of additional arabinose (B). All reactions contained 2.65 µM enzyme (0.1 µg µL^{-1}), 1x RP and 1 mM $CaCl_2$. After adding pNPA to a final concentration of 1 mM the reactions were incubated for 20 min at the respective temperature optimum. Bars represent standard deviation from triplicates

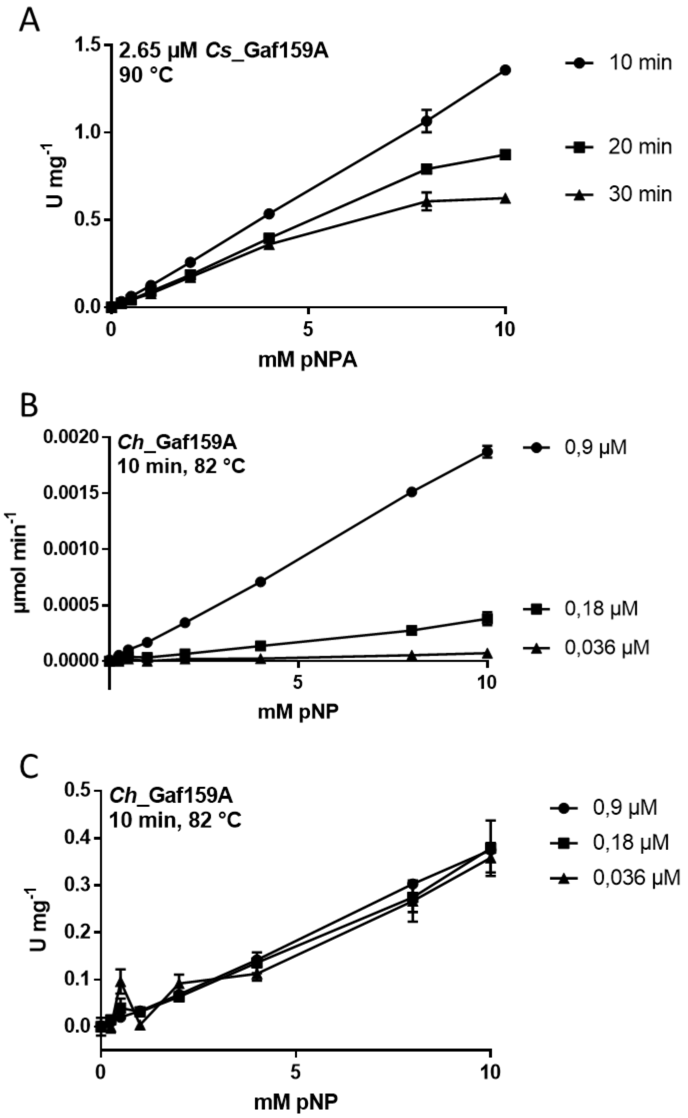

Figure S 4. Attempt to determine kinetic parameters using pNPA. (A) Specific activities of Cs_Gaf159A at different pNPA concentrations. Differences in specific activity between measurements after 10, 20 and 30 min indicate loss in activity over time. (B, C) Activity of Ch_Gaf159A on pNPA, showing pNP production rates (B) and specific activities (C) with enzyme concentrations as indicated. Error bars represent standard deviation of triplicates calculated by Graphpad.

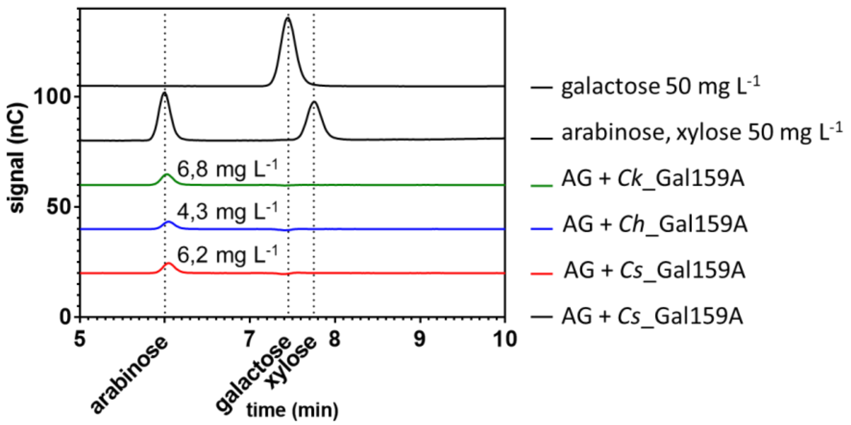

Figure S 5. HPAEC-PAD chromatograms of arabinogalactan degradation with Cs-, Ch- and Ck_Gaf159A. Reactions with 5 g L^{-1} arabinogalactan, 2.65 µM (0.1 g L^{-1}) enzyme, and 1x RP were incubated for 23 h at 80 °C and separated by HPAEC. Galactose, arabinose and xylose with 50 mg L^{-1} each serve as external standard.

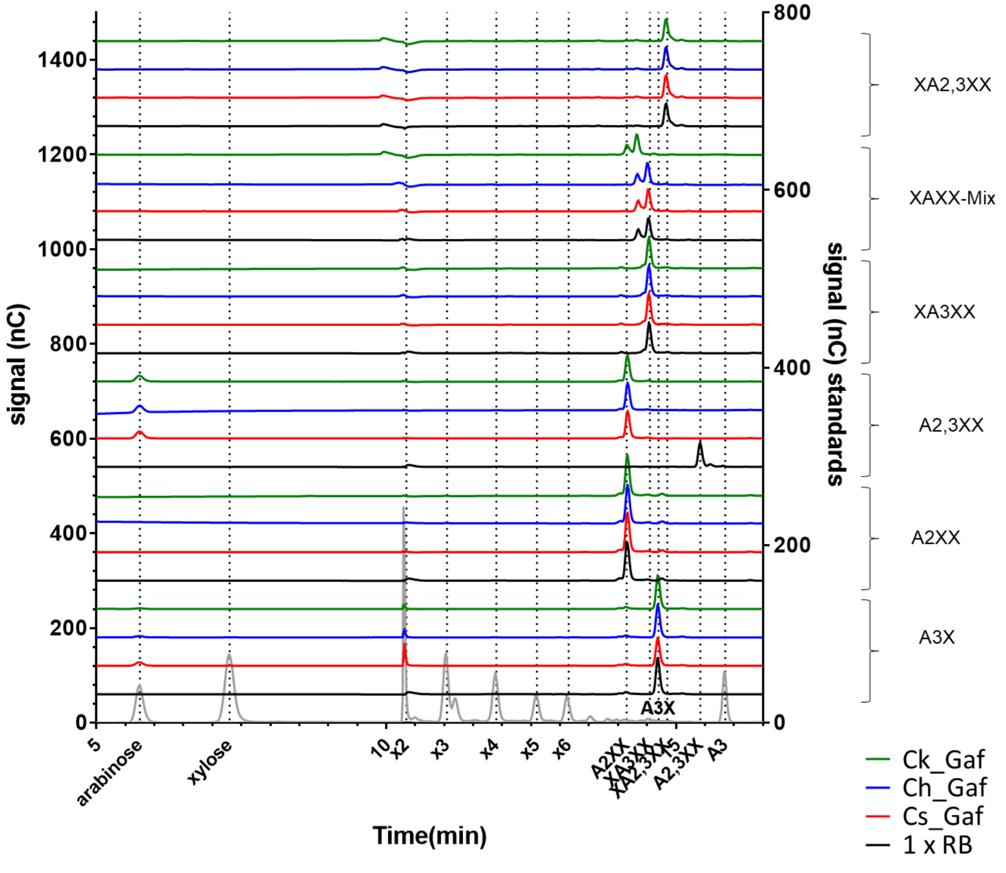

Figure S 6. HPAEC-PAD chromatograms after hydrolysis of different AXOS using Cs_Gaf159A, Ch_Gaf159A and Ck_Gaf159A. Reactions contained 0.1 g L^{-1} substrate (A^3X, A^2XX, $A^{2,3}XX$, XA^3XX, XAXX-Mix or $XA^{2,3}XX$ - nomenclature as described in Material and methods), 0.2 µM (Cs), or 0.1 µM Ch, Ck) enzyme, 1 x RP, 1 mM CaCl2 and were incubated 24 h at 75 °C. A complete conversion from $A^{2,3}XX$ to A^2XX, and thus the release of arabinose, was observed for all three enzymes. Small amounts of arabinose and xylobiose (X2) were produced of A3X by Cs_Gaf159A and Ch_Gaf159A, and even less by Ck_Gaf159A. A^2XX, XA^3XX, XAXX-mix and $XA^{2,3}XX$ were not cleaved by the three enzymes.

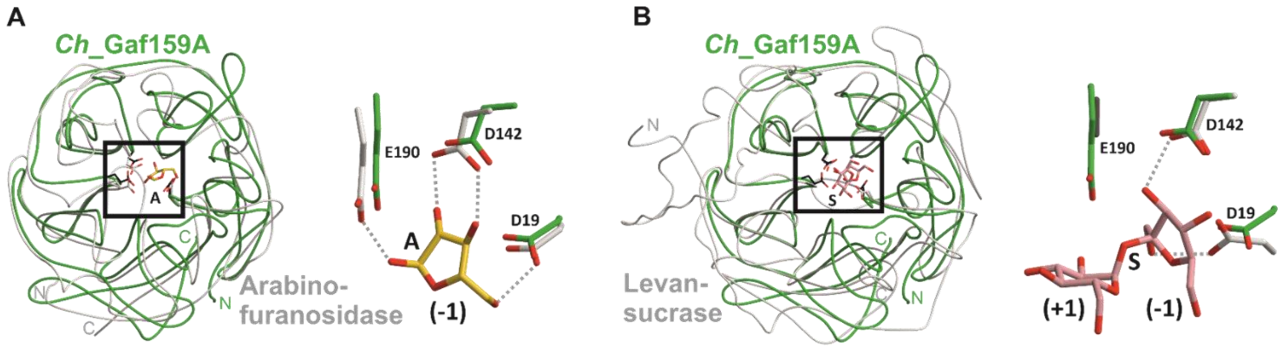

Figure S 7. Structure alignments. (**A**) Superposition of *Ch*_Gaf159A (green) and aribinofuranosidase (grey) in complex with arabinose (A, gold). Close up view of the active site (black rectangle). The ligand at position (-1) is fixed by an extensive hydrogen bonding network (rmsd: 1.6 Å for 143 C_α atoms, sequence identity (SI): 12%, PDB ID: 4O8O) (Wang et al. 2014). (**B)** Superposition of *Ch*_Gaf159A and levansucrase (grey) in complex with sucrose (S, pink). The introduced E342A mutation (dark grey) abolishes hydrolytic enzyme activity. The ligand is bound to the (-1) and (+1) specificity pockets (rmsd: 1.5 Å for 143 C_α atoms, sequence identity (SI): 13%, PDB ID: 1PT2) (Meng and Fütterer 2003).

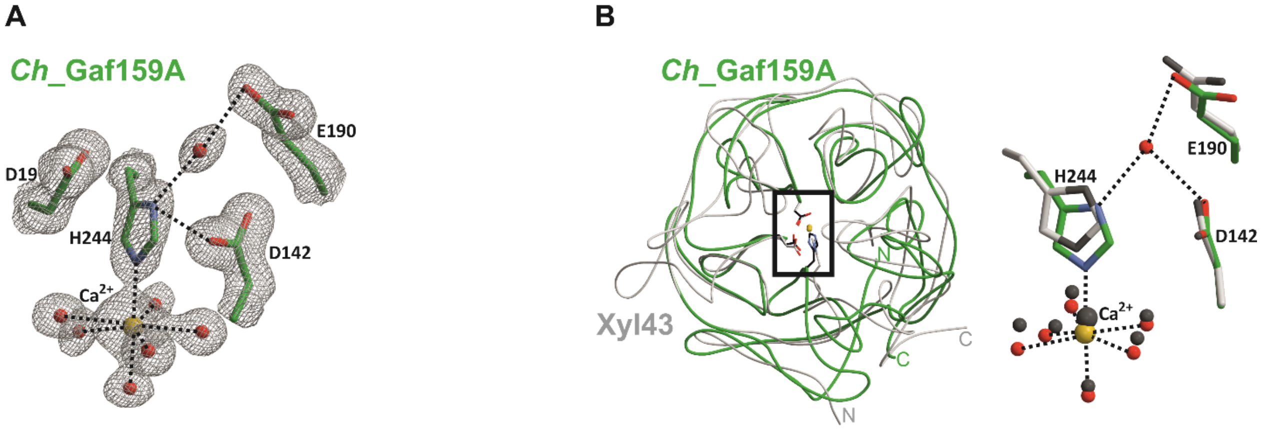

Figure S 8. Active site architecture and Ca2+ coordination.(**A**) Putative residues and a water molecule involved in Gaf159A catalysis are shown in ball-and-stick presentation. In addition, the structure depicts a well-defined metal ion assigned to calcium (Ca2+) that is coordinated by His244 as well as six water molecules (red spheres). The 2Fo-Fc electron density map is contoured to 1σ. (**B**) Structural superposition of Gaf159A and Ca2+ activated GH43 xylosidase (CoXyl43, grey, rmsd: 1.5 Å for 150 Cα atoms, sequence identity: 18%, PDB ID: 1GLR) (Matsuzawa et al. 2017).

```
Q ss_pred                cccCCCCC--CCCCcEEEEECCCCeEEEEEecCcccccCCCCcccCCceEEEEEEeCCCCCCcEeCcEEEcc----CCCCC
Q Q_1703325         8    LFRDPIYD--GAADPTIIYNHLEKSWWILYTNRRANQKLPGKAFMHGTDIGIAESKDGGRTWFYRGTIELQ----YGRGR   81
Q Consensus         8    ~~~~p~~~--~~~dp~v~~~~~~g~~~~~~~~~~~~~~~~~~~~~~~~~i~~~~S~Dlg~~W~~~~~~~~~----~~~~~   81
                         ...||++.  .+.||.+++.  +++|||++....            ...+.+++|+|+ ++|+..+.++..    ..+..
T Consensus         4    ~~~nPv~~~~~~~dp~v~~~--~~~~~~~~~~~~~------------~~~i~~~~S~d~-~~W~~~~~~~~~~~~~~~~~~   68
T 4QQS_B            4    TYTNPVGGITGIGDPYVLKH--ESRYYLYATSAI------------NRGFKVWESPNL-VDWELKGLALDSYYEKNGWGT   68
T ss_dssp               EECSSCTTCCSBEEEEEEEE--TTEEEEEEECCT------------TTEEEEEEESSS-SSCEEEEEEEETTSTTCCCCC
T ss_pred                cccCCCCCCCCCCcEEEEe--CCEEEEEEeCCc------------CCceEEEEeCCC-CCcEEeceecchhhhcCCCCC
Confidence               45688855  6999999999  999999999753            346889999996 999988776542    23344

Q ss_pred                ceEEeEEEEEEECCEEEEEEEcCCCCcccCCeEEEEEEEeCCCCC-CEEeceeecC-CCCeEeEEEEEcCCCcEEEEEEE
Q Q 1703325         82   NTFWAPEVIFYEGEYHMYVSFVPGVPQDWNAERYILYYKSKNLWD-WEFVCKLELS-SNKVIDACVFQMPDGTFRMWYKD  159
Q Consensus        82   ~~~~~p~v~~~~g~~~l~~~~~~~~~~~~~~~~~~i~~a~s~d~~~-w~~~~~~~~~-~~~~~d~~v~~~~~g~~~l~~~~  159
                         ..+++|.+++.+++|+|+|+.....     ....++++.|.++.+ |+........ .....+|.++...+++++|+|..
T Consensus        69   ~~~~~p~v~~~~g~~~~~~y~~~~~-----~~~~i~~a~s~d~~~~~~~~~~~~~~~~~~~~~~~dp~v~~~~~g~~~~~~~~~  143
T 4QQS_B           69   EDFWAPEVIFYNNKFYMTYSARDND-----GHLKIALASSKSPLGPFKNIKAPLFDRGLSFIDAHIFIDQDGTPYIYYVK  143
T ss_dssp               SCCEEEEEEEEETTEEEEEEEECTT-----SCEEEEEEEEESSTTCCCEEEEESSSCCCSSCEEEEEECTTSCEEEEEEE
T ss pred                ccceeeEEEEECCEEEEEEEEECCC-----CCEEEEEEEecCCCCCCccccCCCccCCCCceeeEEEECCCCCEEEEEEE
Confidence               5788999999999999999986532     245789999999887 7765543332 34678999998788999999988

Q ss_pred                CCCC--------eEEEEEE-cCCCCCcEEecccC-CC-----------CCCcccEEEEECCEEEEEEcCC------CcEE
Q Q_1703325        160   EADH--------SYIYAAE-SNNLKDWKILGPAL-TD-----------RPQEGPNVFWWKSKYWMITDPW------CGLG  212
Q Consensus       160   ~~~~--------~~i~~~~-s~d~~~w~~~~~~~~-~~-----------~~~~~p~~~~~~g~~~l~~~~~------~~~~  212
                         ....        ..+.++. +.++..|......+ ..           ...++|.+++.++.|||+|...      ..+.
T Consensus       144   ~~~~~~~~~~~~~~~~~~~~~~s~~~~~~~~~~~~~~~~~~~~~~~~~~~~~~~~~~~p~v~~~~g~~~l~~~~~~~~~~~~~~~~i~  223
T 4QQS_B          144   DCSENIINGIHISQIYVQEMSQDLLELKGDPVLAIQPSQDWEGINDAWQWNEGPFVIKHEGKYYMMYSANCYASPDYSIG  223
T ss_dssp               CTTTCEETTEEEEEEEEEEBCTTSSCBCSCCEEEECSCSGGGCTTSSEEEEEEEEEEEETTEEEEEEEESCTTSTTCEEE
T ss_pred                cCCcCeecCCccceeEEEEeCCccccccCCcEEEeCCCCCcccccceeeEEceEEEEECCEEEEEEEEecCCCCCCeEEE
Confidence               6431        3445554 66677776433221 11           2357799999999999999865      3689

Q ss_pred                EEEcCCCC-Ccee-c-CceecCCCCCccCCCceeceEEEEECCE-EEEEEEEeecCcCCCChhhhcCccceeeEEEEEEE
Q Q_1703325        213   VYSSEDAT-AWHR-H-ENILDRPGKREDDGQIGHHADVLVIDDE-TAYIFYFTHPEGMEGTEEFWKDSKYWRTSLQVAKL  288
Q Consensus       213   ~~~S~d~~-~w~~-~-~~~~~~~~~~~~~~~~~~~~~~~~~~~~-~~~~~y~~~~~~~~~~~~~~~~~~~~~~~~~~~~~~l  288
                         ++.|.++. +|+. . .+++....  .......+++.++...++ .++++|++......       ....+...  +.++
T Consensus       224   ~~~s~d~~~~w~~~~~~~~~~~~~~~--~~~~~~~~~~~~~~~~~~~g~~~~~~~y~~~~~~~~~~-------~~~~~~~~--~~~~  292
T 4QQS B          224   YAVAETPLGPWIKYSGNPILSKRM--DKGISGPGHNSVTVSPDGSELFVVYHTHTYPDS-------PGGDRTVN--IDRL  292
T ss_dssp               EEEESSTTCCCEECTTCCSBCCBG--GGTBCCCEEEEEEECTTSSSEEEEEEEESCTTS-------CSSCEEEE--EEEE
T ss_pred                EEEECCCCCCCcccCCCceeeEeC--CCCeeccccceEEECCCCCCEEEEEEEEcCCCC-------CCCCceEE--EEEE
Confidence               99999987 5986 2 55555432  223345667777775443 38899998764321       22346666  6699

Q ss pred                EEECCEEEEecCCC
Q Q_1703325        289   EYVDGKVVCDRDK  301 (311)
Q Consensus       289   ~~~~~~~~~~~~~  301 (311)
                         .|++|.+.+....
T Consensus       293   ~~~~~~~~~~~~~  305 (315)
T 4QQS B          293   YFEDGILKVKGPT  305 (315)
T ss_dssp               EEETTEEEEECCC
T ss_pred                EEECCEEEEeeCCC
Confidence               9988888877653
```

Figure S 9. Structural alignment by HHpred using ADQ06027.1 (Ch_Gaf159A) as query sequence and default parameters). Amino acids (aa) are highlighted as follows: grey, aa which can be found in the active site of HoAraf43; red, catalytical aa as described by Hassan 2015; green, identical active-site-aa that can be found in the same position in Ch_Ara159A; yellow, similar active-site-aa that can be found in the same position in Ch_Ara159A; purple, aa which are not the same as in HoAraf43. 4QQS_B Glycoside hydrolase family 43; 5-bladed beta-propeller, glycoside hydrolase, hydrolase; HET: EPE; 1.1A {Halothermothrix orenii}; Related PDB entries: 4QQS_A. Probab=99.90 E-value=5.4e-20 Score=150.10 Aligned_cols=263 Identities=24% Similarity=0.348 Sum_probs=187.3 Template_Neff=12.100

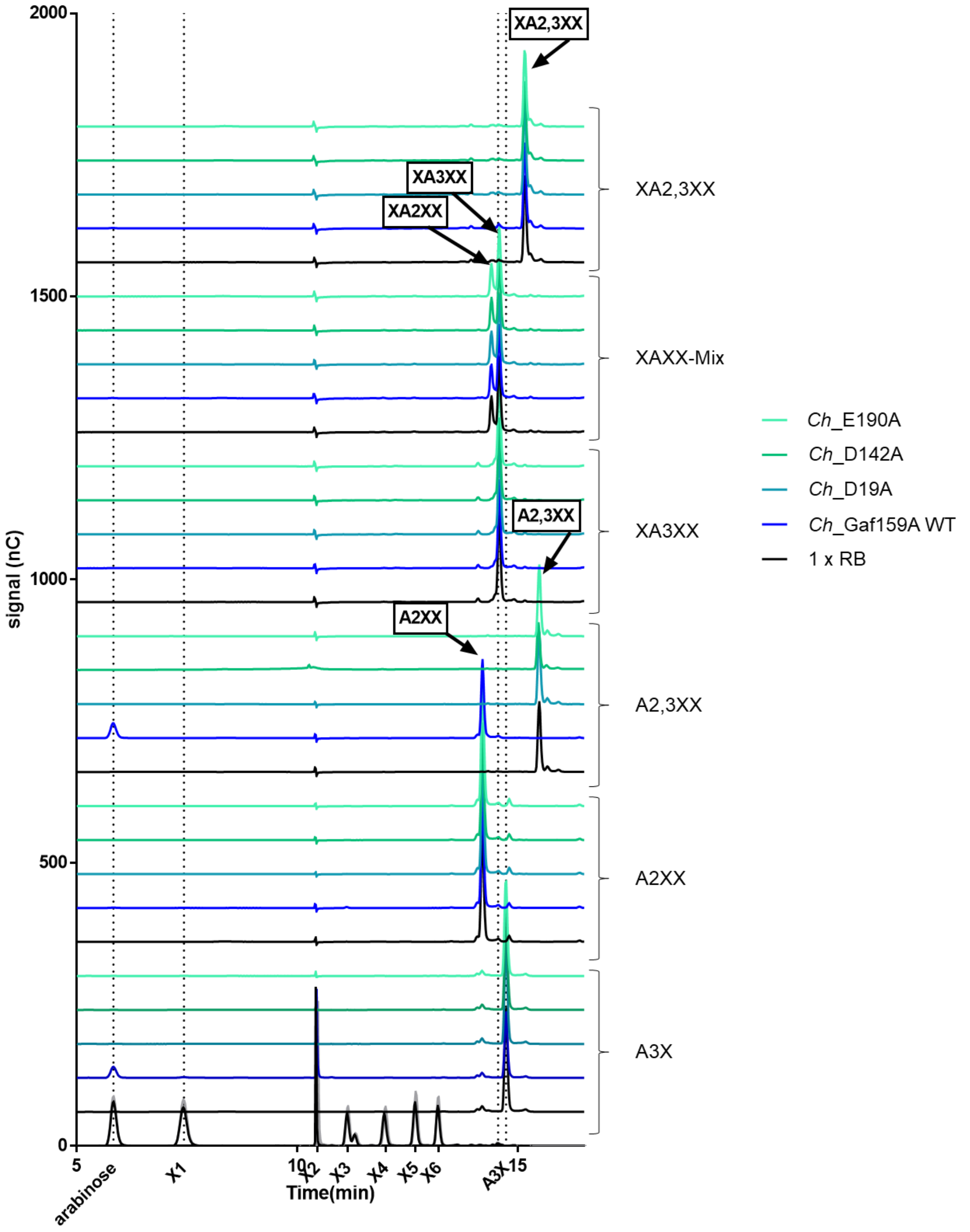

Figure S 10. AXOS degradation by Ch_Gaf159A WT, Ch_D19A, Ch_D142A or Ch_E190A. HPAEC-PAD chromatograms of reactions which were incubated 22 h at 75 °C and contained 0.01% of different AXOS, 1 mM $CaCl_2$, 1x RB and 2.65 µM (0.1 g L^{-1}) of the respective enzymes.

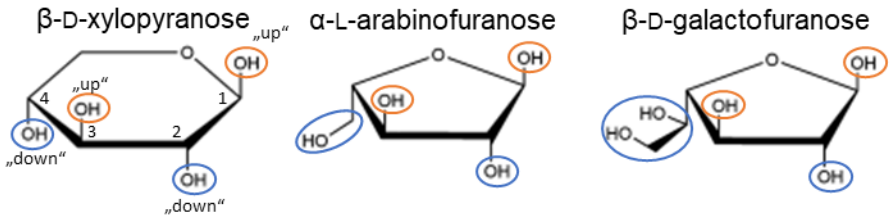

Figure S 11. Similarity of ring substitutions in β-D-xylopyranose, α-L-arabinofuranose and β-D-galactofuranose. Structures are shown in Haworth projection obtained from (McGill and Westmoreland 2018) and were processed to mark the identical orientations.

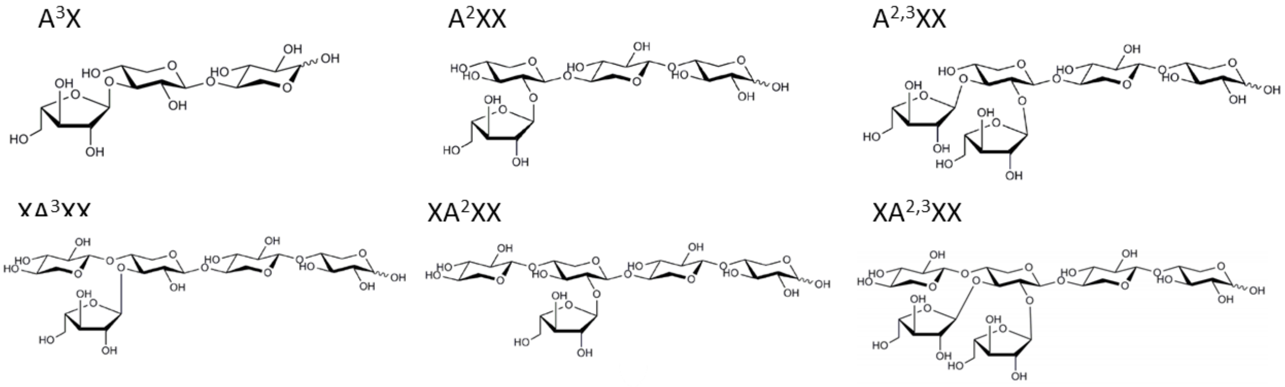

Figure S 12. Structures of AXOS used in this study. Structures obtained from Megazyme datasheets, designated as described in material and methods.

2 Supplementary Tables

Table S 1. Background of salt solutions at 405 nm for metal ion dependency tests. Reactions contained 1 mM pNPA, 1 x RB and additional salts in concentrations as indicated and were incubated for 20 min at the respective temperatures. Mean and standard deviation calculated from triplicates.

	A 405 nm											
	82 °C				90 °C				93 °C			
	1 mM additional solution		10 mM additional solution		1 mM additional solution		10 mM additional solution		1 mM additional solution		10 mM additional solution	
	mean	SD	mean	SD	mean	SD	mean	SD	mean	SD	mean	SD
NaCl	0.041	0.001	0.041	0.001	0.046	0.001	0.046	0.001	0.054	0.003	0.055	0.001
KCl	0.039	0.001	0.040	0.001	0.044	0.003	0.045	0.003	0.051	0.002	0.053	0.001
$CaCl_2$	0.039	0.000	0.043	0.002	0.045	0.003	0.048	0.001	0.052	0.001	0.064	0.002
$CuCl_2$	0.111	0.009	0.043	0.002	0.082	0.011	0.064	0.002	0.116	0.016	0.071	0.010
$MgCl_2$	0.040	0.001	0.042	0.001	0.047	0.001	0.045	0.001	0.053	0.003	0.054	0.001
$MnCl_2$	0.042	0.001	0.374	0.029	0.048	0.001	0.301	0.026	0.060	0.003	0.762	0.053
$ZnCl_2$	0.041	0.002	0.050	0.002	0.048	0.002	0.059	0.003	0.054	0.001	0.070	0.003
H_20	0.041	0.001	0.041	0.001	0.048	0.002	0.048	0.002	0.056	0.002	0.055	0.001
NiSO4	0.043	0.001	0.149	0.008	0.049	0.000	0.137	0.004	0.055	0.002	0.163	0.012
$CoCl_2$	0.040	0.001	0.134	0.003	0.046	0.001	0.131	0.008	0.051	0.002	0.122	0.004
H_2O	0.040	0.001	0.042	0.001	0.046	0.001	0.047	0.001	0.051	0.001	0.050	0.001

Table S 2. pH values of McIlvaine+ and MOPS buffers used for pH optima determination at RT and 80 °C.

**McIlvaine +**		**0,1 M MOPS**	
pH at RT	pH at 80 °C	pH at RT	pH at 80 °C
4.5	4.7	6	5.3
5.0	5.1	6.2	5.5
5.5	5.6	6.4	5.6
6.0	6.0	6.6	5.8
6.5	6.5	6.8	6.0
7.0	6.7	7.0	6.2
7.5	6.9	7.2	6.4
8	7.2	7.4	6.6

Table S 3. X-ray data collection and refinement statistics.

	***Ch*_Gaf159A** from *Caldicellulosiruptor hydrothermalis*
**Crystal parameters**	
Space group	$P2_12_12_1$
Cell constants	a = 91.1 Å
	b = 108.7 Å
	c = 251.3 Å
Molecules / AU[a]	6
**Data collection**	
Beam line	X06SA, SLS
Wavelength (Å)	1.0
Resolution range (Å)[b]	30-1.7 (1.8–1.7)
No. observations	1287141
No. unique reflections[c]	269815
Completeness (%)[b]	98.7 (98.9)
R_{merge} (%)[b, d]	5.7 (51.4)
I/σ (I)[b]	15.5 (2.8)
**Refinement (REFMAC5)**	
Resolution range (Å)	30–1.7
No. refl. working set	256285
No. refl. test set	13488
No. non hydrogen	17777
Solvent molecules	2210
R_{work}/R_{free} (%)[e]	12.8 / 15.3
r.m.s.d. bond (Å) / angle (°)[f]	0.004 / 1.2
Average B-factor ($Å^2$)	22.7
Ramachandran Plot (%)[g]	96.3 / 3.7 / 0
PDB accession code	7ZEI

[a] Asymmetric unit
[b] The values in parentheses for resolution range, completeness, R_{merge} and I/σ (I) correspond to the highest resolution shell
[c] Data reduction was carried out with XDS and from a single crystal. Friedel pairs were treated as identical reflections
[d] $R_{merge}(I) = \Sigma_{hkl}\Sigma_j \mid I(hkl)_j - \langle I(hkl)\rangle \mid / \Sigma_{hkl} \Sigma_j I(hkl)_j$, where $I(hkl)_j$ is the jth measurement of the intensity of reflection hkl and <I(hkl)> is the average intensity
[e] $R = \Sigma_{hkl} \mid |F_{obs}| - |F_{calc}| \mid / \Sigma_{hkl} |Fobs|$, where R_{free} is calculated without a sigma cut off for a randomly chosen 5% of reflections, which were not used for structure refinement, and R_{work} is calculated for the remaining reflections
[f] Deviations from ideal bond lengths / angles
[g] Percentage of residues in favoured region / allowed region / outlier region

Table S 4. Sequence comparison of Cs_Gaf159A, Ch_Gaf159A, Ck_Gaf159A and Ch_Gaf159A mutants D19A, D142A and E190A. Only amino acid positions are shown in which the six enzymes differ. The common one letter code for amino acids was used and hydrophilic residues are highlighted in grey.

	**residue in**					
**position**	***Cs*_**	***Ch*_**	***Ck*_**	***Ch*_D19A**	***Ch*_D142A**	***Ch*_E190A**
3	K	R	R	R	R	R
65	K	R	R	R	R	R
68	L	L	F	L	L	L
74	D	D	E	D	D	D
92	C	Y	Y	Y	Y	Y
112	W	A	A	A	A	A
113	E	D	E	D	D	D
130	V	I	V	I	I	I
131	K	C	C	C	C	C
133	I	L	L	L	L	L
134	D	K	E	K	K	K
162	N	D	D	D	D	D
163	N	H	H	H	H	H
168	V	V	A	V	V	V
172	K	T	N	T	T	T
178	N	K	K	K	K	K
179	V	V	I	V	V	V
196	F	Y	W	Y	Y	Y
199	G	S	S	S	S	S
215	F	S	S	S	S	S
220	N	N	T	N	N	N
221	I	V	A	V	V	V
223	Y	H	H	H	H	H
225	Q	Q	H	Q	Q	Q
231	K	K	R	K	K	K
276	N	N	S	N	N	N
277	R	R	K	R	R	R
291	I	V	V	V	V	V
293	G	D	G	D	D	D
294	K	R	K	R	R	R
295	I	V	V	V	V	V
298	D	Y	D	Y	Y	Y
306	C	Y	Y	Y	Y	Y
309	N	D	D	D	D	D
19	D	D	D	A	D	D
142	D	D	D	D	A	D
190	E	E	E	E	E	A
number of shown hydrophobic residues	23	24	24	25	25	25
number of shown hydrophilic residues	14	13	13	12	12	12

3 Supplementary References

Matsuzawa, Tomohiko; Kaneko, Satoshi; Kishine, Naomi; Fujimoto, Zui; Yaoi, Katsuro (2017): Crystal structure of metagenomic β-xylosidase/ α-l-arabinofuranosidase activated by calcium. In *The Journal of Biochemistry* 162 (3), pp. 173–181. DOI: 10.1093/jb/mvx012.

McGill, Charles J.; Westmoreland, Phillip R. (2018): Monosaccharide isomer interconversions become significant at high temperatures. In *The Journal of Physical Chemistry A* 123 (1), pp. 120–131.

Meng, Guoyu; Fütterer, Klaus (2003): Structural framework of fructosyl transfer in Bacillus subtilis levansucrase. In *Nature structural biology* 10 (11), pp. 935–941. DOI: 10.1038/nsb974.

Wang, Weijun; Mai-Gisondi, Galina; Stogios, Peter J.; Kaur, Amrit; Xu, Xiaohui; Cui, Hong et al. (2014): Elucidation of the molecular basis for arabinoxylan-debranching activity of a thermostable family GH62 α-l-arabinofuranosidase from Streptomyces thermoviolaceus. In *Appl Environ Microbiol* 80 (17), pp. 5317–5329. DOI: 10.1128/AEM.00685-14.
